# Colorectal Cancer‐Derived Small Extracellular Vesicles Promote Tumor Immune Evasion by Upregulating PD‐L1 Expression in Tumor‐Associated Macrophages

**DOI:** 10.1002/advs.202102620

**Published:** 2022-01-17

**Authors:** Yuan Yin, Bingxin Liu, Yulin Cao, Surui Yao, Yuhang Liu, Guoying Jin, Yan Qin, Ying Chen, Kaisa Cui, Leyuan Zhou, Zehua Bian, Bojian Fei, Shenglin Huang, Zhaohui Huang

**Affiliations:** ^1^ Wuxi Cancer Institute Affiliated Hospital of Jiangnan University Wuxi 214062 China; ^2^ Laboratory of Cancer Epigenetics Wuxi School of Medicine Jiangnan University Wuxi 214122 China; ^3^ Department of Pathology Affiliated Hospital of Jiangnan University Wuxi Jiangsu 214062 China; ^4^ Department of Radiation Oncology Affiliated Hospital of Jiangnan University Wuxi 214062 China; ^5^ Department of Gastrointestinal Surgery Affiliated Hospital of Jiangnan University Wuxi 214062 China; ^6^ Fudan University Shanghai Cancer Center and Institutes of Biomedical Sciences Fudan University Shanghai 200032 China

**Keywords:** colorectal cancer, macrophage, MicroRNA, PD‐L1, small extracellular vesicles

## Abstract

Tumor‐associated macrophages (TAMs) are one of the most abundant cell types in colorectal cancer (CRC) tumor microenvironment (TME). Recent studies observed complicated “cross‐talks” between cancer cells and macrophages in TME. However, the underlying mechanisms are still poorly elucidated. Here, PD‐L1 levels are very low in CRC cells but highly abundant in TAMs, and a specific PD‐L1^+^CD206^+^ macrophage subpopulation are identified, which is induced by tumor cells and associated with a poor prognosis. Mechanistic investigations reveal that CRC cells can secrete small extracellular vesicles (sEVs) taken up by macrophages that induce M2 like polarization and PD‐L1 expression, resulting in increased PD‐L1^+^CD206^+^ macrophage abundance and decreased T cell activity in CRC TME. sEV‐derived miR‐21‐5p and miR‐200a are identified as key signaling molecules mediating the regulatory effects of CRC on macrophages. Further studies reveal that CRC‐derived miR‐21‐5p and miR‐200a synergistically induces macrophage M2 like polarization and PD‐L1 expression by regulating the PTEN/AKT and SCOS1/STAT1 pathways, resulting in decreased CD8^+^ T cell activity and increased tumor growth. This study suggests that inhibiting the secretion of specific sEV‐miRNAs from CRC and targeting PD‐L1 in TAMs may serve as novel methods for CRC treatment as well as a sensitization method for anti‐PD‐L1 therapy in CRC.

## Graphical abstract

A specific PD‐L1^+^CD206^+^ macrophage risk subgroup is identified in colorectal cancer, which is induced by tumor‐derived sEV‐miRNAs. This subgroup promotes tumor growth by inducing an immunosuppressive tumor microenvironment. Hence, targeting specific tumor sEV‐miRNAs that regulate PD‐L1 in tumor‐associated macrophages may serve as a novel treatment strategy and a sensitization method for anti‐PD‐L1 therapy in colorectal cancer.

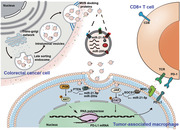

## 1 Introduction

Colorectal cancer (CRC) is one of the most common cancers worldwide.^[^ ^1^ ^]^ CRC development and progression are not only controlled by genetic and epigenetic regulation but also closely related to the tumor microenvironment (TME), especially the tumor immune microenvironment. In the past decade, immune checkpoint blockade (ICB) has attracted great attention for its promising efficacy in the treatment of malignant solid tumors, such as melanoma and non‐small cell lung cancer.^[^ ^2^ ^]^ ICB therapy is also approved for the treatment of CRC patients with DNA mismatch repair‐deficient (dMMR)/MSI‐H molecular characteristics. However, the dMMR/MSI‐H CRC subgroup accounts for only 15% of CRC patients, and most CRC patients do not benefit from ICB treatment.^[^ ^3^ ^]^ Therefore, investigating the interaction between CRC and immune system, then modulating immune cells in the TME, may provide an effective strategy for the immunotherapy of CRC.

Macrophages are one of the most abundant immune cell types present in the TME, in which they are termed tumor‐associated macrophages (TAMs). We previously demonstrated that TAMs increase CRC chemoresistance and inhibit drug‐induced apoptosis by secreting IL6, which regulates the STAT3‐miR‐204 axis in CRC cells.^[^ ^4^ ^]^ Other groups have also reported that PD‐1 expressed by TAMs inhibits phagocytosis and antitumor immunity^[^ ^5^ ^]^ and that TAM‐derived CCL5 facilitates immune escape of CRC cells via the p65/STAT3‐CSN5‐PD‐L1 pathway.^[^ ^6^ ^]^ These studies suggest that TAMs can suppress local immunity and contribute to tumor immune escape and progression, and TAMs may represent a potential targets in cancer immunotherapy. However, how TAMs are educated in the CRC TME remain elusive.^[^ ^7^ ^]^

Small extracellular vesicles (sEVs) are cell‐secreted extracellular vesicles with a diameter of 30–150 nm that have emerged as important regulators of intercellular signaling^[^ ^8^ ^]^ and promising biomarkers.^[^ ^9^ ^]^ sEVs carry complex biological molecules, including proteins, lipids, DNA and RNA.^[^ ^10^ ^]^ Among these cargoes, microRNAs (miRNAs) are considered to be one of the most important signaling molecules: 1) miRNAs are 21–25 nucleotides in length and can be efficiently packaged into sEVs; 2) miRNAs are stably distributed in various tissues and body fluids; and 3) a single miRNA can regulate multiple target genes, while, conversely, multiple miRNAs may act synergistically to regulate a single gene, leading to an efficient and flexible gene regulation pattern.^[^ ^11^ ^]^ We^[^ ^12^ ^]^ and other groups^[^ ^13^ ^]^ have confirmed that sEV‐mediated delivery of functional miRNAs plays key roles in intercellular communication in the TME.

**Scheme 1:**
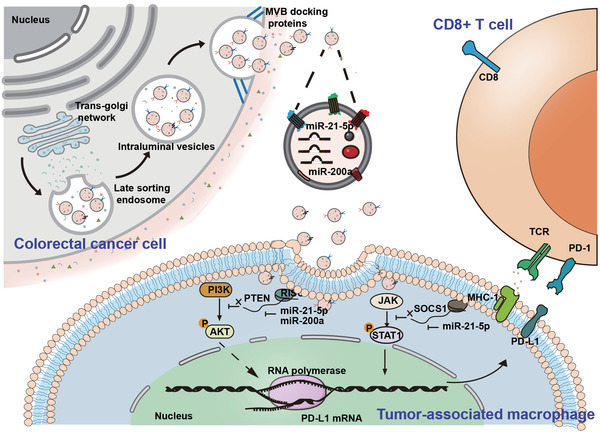
Schematic illustration of CRC‐derived sEVs enhance PD‐L1 expression in TAMs, then suppresses CD8^+^ T cells in TME and promotes CRC tumor growth.

In this study, we observed that PD‐L1 levels are very low in CRC cells but highly abundant in TAMs, and identified a PD‐L1^+^CD206^+^ macrophage subpopulation, which was induced by tumor cells during the course of macrophage infiltration and associated with a poor prognosis. Detailed mechanism investigations revealed that CRC‐derived sEV‐miR‐21‐5p and sEV‐miR‐200a synergistically induced TAM M2 like polarization and to express PD‐L1 through the PTEN/AKT and SOCS1/STAT1 signaling pathways, promote TAM‐mediated inhibition of CD8^+^ T lymphocytes, and thus contribute to immune escape and CRC progression. We demonstrates how CRC cells acclimate macrophages to help tumor cells achieve immune escape through secretion of multiple sEV‐miRNAs, which provides a new target for CRC immunotherapy (**Scheme** **1**).

## 2 Results

### 2.1 Alteration of Macrophage‐Associated Immunity in the CRC TME

**Figure 1:**
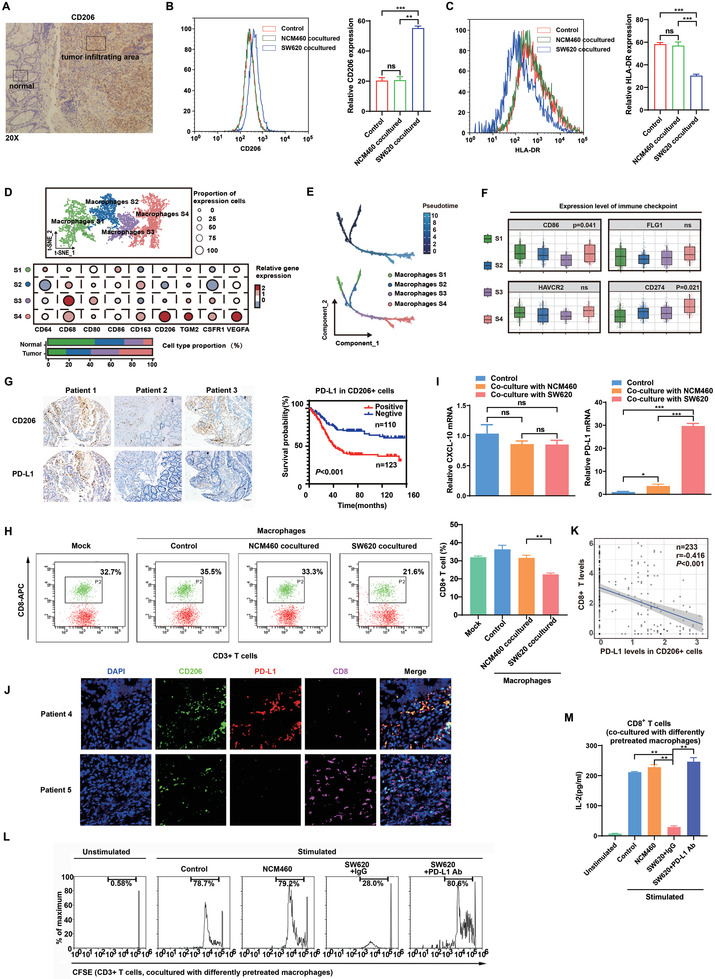
Alteration of macrophage‐associated immunity in the CRC TME. A) IHC staining of CD206^+^ macrophages in CRC tissues. B, C) THP‐1‐derived macrophages were cocultured with NCM460 or SW620 cells for 48 h, and flow cytometry was then performed to detect the expression of B) CD206 and C) HLA‐DR in these macrophages. D) Molecular characteristics of macrophages with different phenotypes in the CRC TME based on the single‐cell sequencing data of GEO datasets (GSE132465, GSE132257, and GSE144753). E) tSNE plot of four subgroups of macrophages using Pseudotime trajectory analyses based on the scRNA‐seq of GEO datasets (GSE132465, GSE132257, and GSE144753). F) Expression of immune checkpoint molecules on the surface of four subgroups of macrophages in the CRC TME (GSE132465, GSE132257, and GSE144735). G) IHC staining of CD68, CD206, and PD‐L1 in 233 paired human CRC tissues and adjacent normal colonic tissues. Survival analysis was performed based on PD‐L1^+^CD206^+^ TAMs. H) Macrophages were cocultured with NCM460 or SW620 cells for 48 h, followed by coculture with human peripheral blood lymphocytes (PBLs) for 72 h. Flow cytometry was performed to detect CD8^+^ T cells. I) CXCL10 and PD‐L1 mRNA expression in THP‐1‐derived macrophages cocultured with NCM460 or SW620 cells was examined by qRT‐PCR. J) The staining of DAPI, CD206, PD‐L1 or CD8 was performed in CRC tissue samples using mIF. K) The negative correlation between the levels of CD8^+^ T cells and PD‐L1 expression in CD206^+^ TAMs in 233 paired CRC tissues. L) Representative histogram of CFSE‐labeled human peripheral CD8^+^ T cells that were cocultured with differently pretreated THP‐1‐derived macrophages. M) IL‐2 levels were detected in the culture supernatants of human peripheral CD8^+^ T cells cocultured with THP‐1‐derived macrophages that underwent different pretreatments by ELISA. * *p* < 0.05, ** *p* < 0.01, *** *p* < 0.001.

We previously demonstrated that TAMs are associated with chemoresistance and poor survival in CRC.^[^ ^4^ ^]^ Here, we further investigated TAM‐associated immunity changes in the CRC TME. IHC staining results showed that the CD206^+^ macrophages density was often higher in the center of tumor tissues than in the tumor margin or in adjacent tissues (**Figure** **1**A), suggesting a phenotypic shift in macrophages when infiltrating CRC tissues. To investigate this hypothesis, we cocultured THP‐1‐derived macrophages with NCM460 or SW620 cells. Macrophages incubated with SW620 cells exhibited a CD206^high^/HLA‐DR^low^ phenotype compared with NCM460 cell‐cocultured or untreated macrophages (Figure 1B,C). To further confirm the phenotypic shift in macrophages in CRC, we isolated primary TAMs from fresh CRC tissues and found that these TAMs secreted significantly more of the tumor‐promoting cytokine IL‐10 and less of the tumor‐suppressing cytokine IL‐12 than freshly isolated human monocytes (Figure S1A,B, Supporting Information). Consistent with these changes in TAMs, macrophages cocultured with SW620 cells also produced more IL‐10 and less IL‐12 than those cocultured with NCM460 cells or left untreated (Figure S1C,D, Supporting Information).

To further investigate how CRC influences the infiltration of TAMs, single‐cell sequencing data were used to map CRC TAMs and reveal their molecular characteristics. Four distinct subgroups of TAMs were clustered (S1–S4), and S4, which exhibited a CD206^high^ phenotype, was found to be elevated in tumors, whereas S1 was found at low levels in tumor sites (Figure 1D). Subsequently, pseudotime trajectory analyses were used to reveal the phenotypic changes in macrophages during the tumor‐mediated education process. We found that with the progression of CRC, TAMs tended to be in the S4 subgroup (Figure 1E). GSEA results showed that the differentially expressed genes of TAMs during the tumor‐mediated education process were enriched in several immunosuppressive signaling pathways (Figure S2A, Supporting Information). Due to the role of macrophages in the immune response, we analyzed the expression of immune checkpoint ligands in CRC TAMs in data from the GEO databases (GSE132465,GSE132257, and GSE144735) and revealed that the S4 subgroup expressed high levels of CD274 (PD‐L1) compared with the other subgroups (Figure 1F).

Based on the TCGA and GEO databases, we found that PD‐L1 expression in CRC tissues was not associated with tumor stage or prognosis. Interestingly, PD‐L1 expression in CD206^+^ macrophages, but not that in CD68^+^ macrophages, was correlated with worse outcomes in CRC, suggesting the prognostic value of PD‐L1^+^CD206^+^ macrophages within the CRC TME (Figure S2B–D). Furthermore, Cox regression analyses showed that the infiltration of CD206^+^PD‐L1^+^ macrophage appears to be an independent risk factor in CRC patients after adjusting other factors such as tumor location and stage (Tables S2 and S3, Supporting Information). This finding was further validated in an independent CRC cohort collected from our hospital. As expected, we observed that CD206^+^ macrophages had significantly higher PD‐L1 levels than other TAM subpopulations, and the abundance of PD‐L1^+^CD206^+^ macrophages in CRC tissues predicted a poor prognosis (Figure 1G). In order to clarify the clinical significance of the TAMs clusters, we also evaluated the correlation between the relative abundance of the CD206^+^PD‐L1^+^TAMs subgroups in patients from TCGA/GEO CRC cohort and single‐cell sequencing cohort with the patient's tumor pathological information, respectively. As shown in Figure S3 in the Supporting Information, the results suggested that there are great differences in the types of TAMs not only in the different tumor locations and adjacent tissues from the same colorectal cancer patient, but also in tumor tissues from patients with different stages. To further investigate the potential relationship between PD‐L1 (CD274) and macrophage subpopulations, we performed additional analyses using two different methods (CIBERSORTx^[^ ^14^ ^]^ and xCell^[^ ^15^ ^]^). The results showed that CD274 expression was significantly correlated with M2 macrophage levels but not M1 macrophage levels in CRC tissues (Figure S4, Supporting Information). Also, we isolated primary macrophages from fresh clinical CRC tissues and detected their expression of CD206 and PD‐L1 by immunofluorescence. Good synergistic expression was observed between CD206 and PD‐L1 in either tumors or adjacent noncancerous tissues (Figure S5, Supporting Information).

In view of the key role of PD‐L1 in regulating T cell activity, we evaluated whether TAMs play immunosuppressive roles in the TME by regulating CD8^+^ T cells. Untreated macrophages and macrophages precultured with NCM460 or SW620 cells were separately cocultured with freshly isolated human lymphocytes. The results showed that macrophages precultured with SW620 cells decreased the CD8^+^ T cell proportion compared with NCM460‐cocultured and untreated macrophages (Figure 1H). To investigate how TAMs impact the CD8^+^ T cell proportion, we detected the mRNA expression of CXCL10 (a key CD8^+^ T cell‐recruiting factor) and PD‐L1, and found that PD‐L1, but not CXCL10, was significantly upregulated in SW620 cell‐precocultured macrophages (Figure 1I). Collectively, these data suggest that PD‐L1‐mediated immunosuppression based on the interaction between macrophages and CD8^+^ T cells may be responsible for the alterations in immunity within the CRC TME.

We then determined whether PD‐L1 expression in macrophages contributes to the immunosuppressive functions of TAMs. The results of mIF staining revealed an inverse correlation between the percentages of PD‐L1^+^CD206^+^ macrophages and CD8^+^ T cells in CRC tissues, suggesting that PD‐L1^+^ CD206^+^ macrophages suppress CD8^+^ T cell infiltration in the TME (Figure 1J). Further IHC staining in 233 CRC tissues and mRNA levels in TCGA database confirmed the negative correlation (Figure 1K; Figure S2E, Supporting Information). In vitro studies showed that macrophages precultured with SW620 cells inhibited the proliferation and IL2 production of CD8^+^ T cells compared with NCM460‐incubated macrophages; these effects were almost totally abolished by anti‐PD‐L1 antibodies (Figure 1L,M). Together, these data demonstrate that PD‐L1 in TAMs functions to suppress CD8^+^ T cells in the CRC TME.

### 2.2 sEVs from CRC Cells Educate Macrophages and Promote PD‐L1 Expression In Vitro

**Figure 2:**
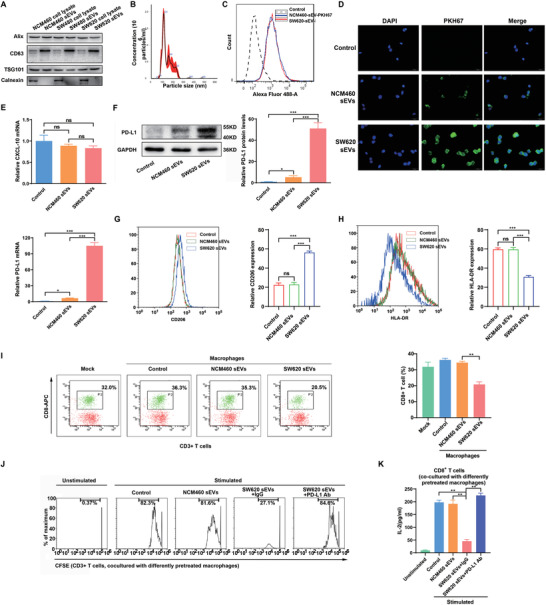
sEVs derived from CRC cells educate macrophages and promote macrophage PD‐L1 expression in vitro. A) Western blot analyses were performed to detect typical sEVs biomarkers (CD63, TSG101 and Alix as positive markers, Calnexin as negative marker) in sEVs derived from CRC cell lines or normal colonic epithelial cells and the corresponding cells. B) Characterization of CRC sEVs by Nanopaticle Tracking Analysis (NTA). C,D) Uptake of PKH67‐labeled NCM460/SW620 cell‐derived sEVs (NCM460/SW620 sEVs, green) by macrophages detected by C) flow cytometry and D) immunofluorescence. E–H) THP‐1‐derived macrophages were cocultured with NCM460 or SW620 sEVs for 48 h. E) CXCL10 and PD‐L1 mRNA levels were examined by qRT‐PCR, and F) PD‐L1 protein levels were examined by western blot. G) Flow cytometry was performed to detect the expression of CD206 and H) HLA‐DR in the macrophages. I) THP‐1‐derived macrophages were cocultured with NCM460 or SW620 sEVs for 48 h, followed by coculture with human peripheral blood lymphocytes for 72 h. Flow cytometry was performed to detect CD8^+^ T cells. J) Representative histogram of CFSE‐labeled human peripheral CD8^+^ T cells that were cocultured with NCM460 or SW620 sEV‐pretreated macrophages. K) IL‐2 levels were detected in the culture supernatant of human peripheral CD8^+^ T cells that were cocultured with NCM460 or SW620 sEV‐pretreated macrophages by ELISA. * *P* < 0.05, ** *p* < 0.01, *** *p* < 0.001.

Our previous study revealed a major link between immune evasion and tumor growth mediated by tumor‐secreted sEVs.^[^ ^4^ ^]^ We speculated that CRC‐derived sEVs are responsible for the modulation of TAMs by CRC. sEVs from NCM460, CCC‐HIE‐2, SW480, SW620, LoVo, and HCT116 cells were verified by western blot and NanoSight tracking analyses (**Figure** **2**A,B; Figure S6A, Supporting Information). The sEV techniques we used were deposited to EV‐TRACK (evtrack.org) with the accession number EV210282.^[^ ^16^ ^]^ Flow cytometric and IF analyses demonstrated that sEVs derived from intestinal cell lines could be efficiently taken up by macrophages (Figure 2C,D). Consistent with the results of the coculture experiments, PD‐L1, but not CXCL10, was significantly upregulated in SW620 sEV‐precocultured macrophages (Figure 2E,F). Consistent with the results of the coculture experiments, macrophages incubated with CRC sEVs exhibited a CD206^high^/HLA‐DR^low^ phenotype compared with control macrophages (Figure 2G,H). Furthermore, the introduction of CRC sEVs significantly repressed the proportion, proliferation and IL‐2 secretion of CD8^+^ T cells (Figure 2I–K). Therefore, CRC cells may educate macrophages and promote macrophage PD‐L1 expression via the delivery of sEVs in vitro.

### 2.3 CRC‐Derived sEVs Educate Macrophages and Promote PD‐L1 Expression In Vivo

**Figure 3:**
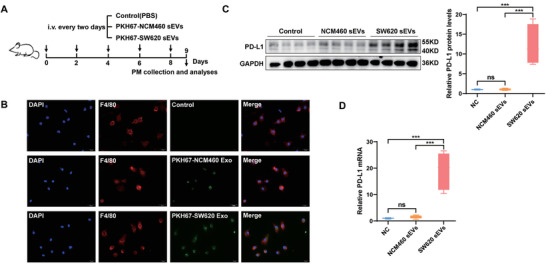
sEVs from CRC cells promote macrophage PD‐L1 expression in vivo. A) Flow chart depicting the experimental design. PKH67‐labeled sEVs from NCM460 (NCM460 sEVs) or SW620 (SW620 sEVs) cells were administered to BALB/c mice via tail vein injection at the same dose (100 µg per 100 µL per mouse) once every 2 days (5 times). The day after the last injection, the mice were sacrificed, and peritoneal macrophages were extracted for subsequent experiments (*n* = 7 for each group). B) Uptake of PKH67‐labeled NCM460 or SW620 sEVs (green) by macrophages detected by immunofluorescence. C,D) PD‐L1 expression in mouse peritoneal macrophages was detected by C) western blotting and D) qRT‐PCR. * *p* < 0.05, ** *p* < 0.01, *** *p* < 0.001.

Given the ability of CRC sEVs to promote PD‐L1 expression in macrophages in vitro, we wanted to determine whether these sEVs can enter and regulate macrophages in vivo. To do so, we administered PKH67‐labeled sEVs from NCM460 or SW620 cells to mice via tail vein injection (**Figure** **3**A). Peritoneal macrophages were isolated from these mice and subjected to IF assays. Consistent with the in vitro results, although both NCM460 and SW620 sEVs efficiently entered macrophages (Figure 3B), SW620 sEVs intensively promoted PD‐L1 expression in the macrophages (Figure 3C,D)

### 2.4 Screening miRNAs Enriched in CRC sEVs

**Figure 4:**
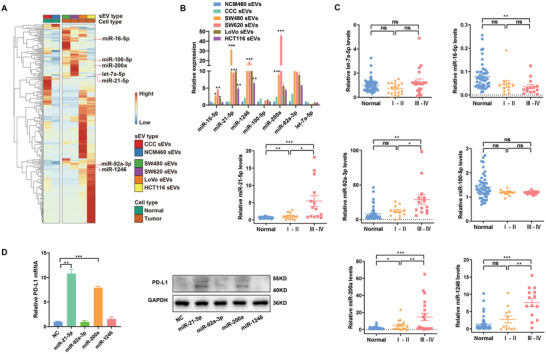
Screening of CRC‐derived sEV‐miRNAs that induce PD‐L1 expression. A) Heatmap of miRNA levels in sEVs derived from CCC‐HIE‐2(CCC), NCM460, SW480, SW620, LoVo, or HCT116 cells. B,C) Candidate miRNAs were validated in sEVs derived from B) CRC cells or plasma samples of C) CR patients using qRT‐PCR. D) The expression levels of PD‐L1 in THP‐1‐derived macrophages transfected with miR‐21‐5p, miR‐92‐3p miR‐1246, and miR‐200a were determined by qRT‐PCR and western blotting. * *p* < 0.05, ** *p* < 0.01, *** *p* < 0.001.

According to our previous study, miRNAs are considered to be one of the most promising cargoes carried by sEVs.^[^ ^12^ ^]^ We hypothesized that CRC‐derived sEV‐miRNAs mediate cancer cell‐macrophage regulation in the TME. sEVs derived from two normal intestinal epithelial cell lines (CCC‐HIE‐2 and NCM460) and four CRC cell lines (SW480, SW620, LoVo, and HCT116) were analyzed by small RNA sequencing (**Figure** **4**A; TableS4, Supporting Information). Seven miRNAs (miR‐16‐5p, miR‐21‐5p, miR‐1246, miR‐100‐5p, miR‐200a, miR‐92‐3p, and let‐7a‐5p) were obviously enriched in CRC sEVs compared with sEVs from normal intestinal epithelial cells.

Next, we validated these upregulated miRNAs using a qRT‐PCR assay and confirmed that miR‐21‐5p, miR‐1246, miR‐200a, and miR‐92a‐3p were significantly upregulated in the sEVs from all the four kinds of CRC cell lines (Figure 4B). To further investigate these sEV‐miRNAs in CRC, we isolated plasma sEVs from CRC patients as well as healthy volunteers. Compared with healthy controls, four miRNAs (miR‐21‐5p, miR‐1246, miR‐200a, and miR‐92a‐3p) were confirmed to be increased in the plasma sEVs of CRC patients, especially those of advanced CRC patients (Stage III–IV) (Figure 4C).

### 2.5 CRC‐Derived sEV‐miR‐21‐5p and sEV‐miR‐200a Educate Macrophages and Synergistically Induce PD‐L1 Expression

**Figure 5:**
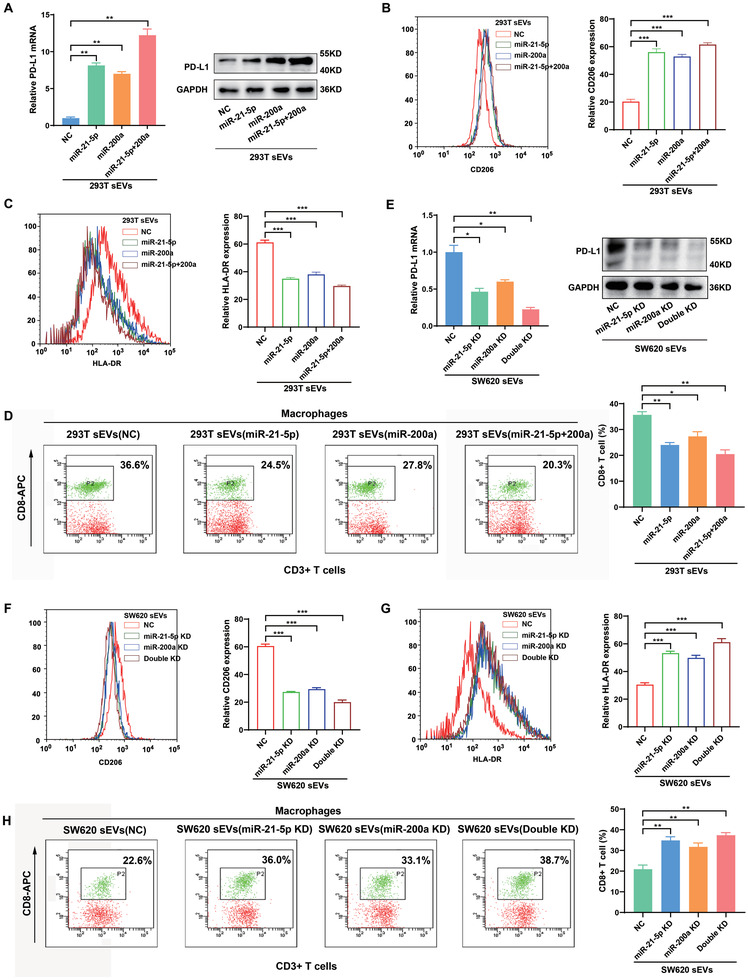
CRC‐derived sEV‐miR‐21‐5p and sEV‐miR‐200a educate macrophages and induce PD‐L1 expression. A) The expression of PD‐L1 in THP‐1‐derived macrophages cocultured with 293T‐sEV‐miR‐21‐5p and/or 293T‐sEV‐miR‐200a were detected by qRT‐PCR and western blotting. B,C) THP‐1‐derived macrophages were cocultured 293T sEV‐miR‐21‐5p and/or 293T sEV‐miR‐200a for 48 h. Flow cytometry was performed to detect the expression of B) CD206 and C) HLA‐DR in the macrophages. D) THP‐1‐derived macrophages were cocultured with 293T sEV‐miR‐21‐5p and/or 293T sEV‐miR‐200a for 48 h, followed by coculture with human peripheral blood lymphocytes for 72 h. Flow cytometry was performed to detect CD8^+^ T cells. E) qRT‐PCR and western blotting were used to determine the expression of PD‐L1 in macrophages treated with sEVs derived from miR‐21‐5p‐ and/or miR‐200a‐silenced SW620 cells. F,G) THP‐1‐derived macrophages were cocultured with sEVs derived from miR‐21‐5p‐ and/or miR‐200a‐silenced SW620 cells for 48 h. Flow cytometry was performed to detect the expression of CD206 and G) HLA‐DR in the macrophages. H) THP‐1‐derived macrophages were cocultured with sEVs derived from miR‐21‐5p‐ and/or miR‐200a‐silenced SW620 cells for 48 h, followed by coculture with human peripheral blood lymphocytes for 72 h. Flow cytometry was performed to detect CD8^+^ T cells. * *p* < 0.05, ** *p* < 0.01, *** *p* < 0.001.

To investigate which miRNAs may be responsible for PD‐L1 upregulation in macrophages, mimics of the four identified miRNAs (miR‐21‐5p, miR‐1246, miR‐200a, and miR‐92a‐3p) were transfected into macrophages separately. As shown in Figure 4D, of the four miRNAs, miR‐21‐5p and miR‐200a significantly increased PD‐L1 expression both at mRNA and protein levels. To further determine whether sEV‐miR‐21‐5p and sEV‐miR‐200a can upregulate PD‐L1 expression in macrophages, we generated 293T cells that stably overexpressed miR‐21‐5p and/or miR‐200a. These cells could efficiently secrete sEVs containing high levels of miR‐21‐5p and/or miR‐200a (Figure S6B,C, Supporting Information). Compared with control 293T sEVs, 293T sEVs with high levels of miR‐21‐5p or miR‐200a significantly increased PD‐L1 expression in macrophages (**Figure** **5**A), and these sEV‐treated macrophages exhibited a CD206^high^/HLA‐DR^low^ phenotype (Figure 5B,C). Consistent with these findings, sEV‐miR‐21‐5p‐ and/or sEV‐miR‐200a‐treated macrophages significantly inhibited the activity of CD8^+^ T cells compared with the control, which were blocked by anti‐PD‐L1 (Figure 5D; Figure S6D,E, Supporting Information).

Loss‐of‐function experiments were performed to further test the effects of CRC‐derived sEV‐miR‐21‐5p and sEV‐miR‐200a on macrophages. We used a “miRNA sponge” technology to produce miR‐21‐5p‐ and miR‐200a‐depleted sEVs (sEV‐miR‐21‐5p KD and sEV‐miR‐200a KD) as we previously described.^[^ ^12^ ^]^ The sponge method efficiently decreased miR‐21‐5p and miR‐200a levels in both SW620 cells and their sEVs (Figure S7A,B, Supporting Information). As shown in Figure 5E, sEVs derived from miR‐21‐5p‐ and miR‐200a‐depleted CRC cells decreased PD‐L1 levels in macrophages, and induced a CD206^low^/HLA‐DR^high^ phenotype of macrophages (Figure 5F,G). Likewise, macrophages pretreated with these CRC sEVs increased the proportion and function of CD8^+^ T cells compared with the control (Figure 5H; Figure S7C,D, Supporting Information). Taken together, these data indicate that CRC‐derived sEV‐miR‐21‐5p and sEV‐miR‐200a could synergistically induce PD‐L1 expression and M2 like polarization in macrophages, resulting in decreasing CD8^+^ T cell activities.

### 2.6 CRC‐Derived sEV‐miR‐21‐5p and sEV‐miR‐200a Induce PD‐L1 Expression in Macrophages through the PTEN/AKT and SCOC1/STAT1 Pathways

**Figure 6:**
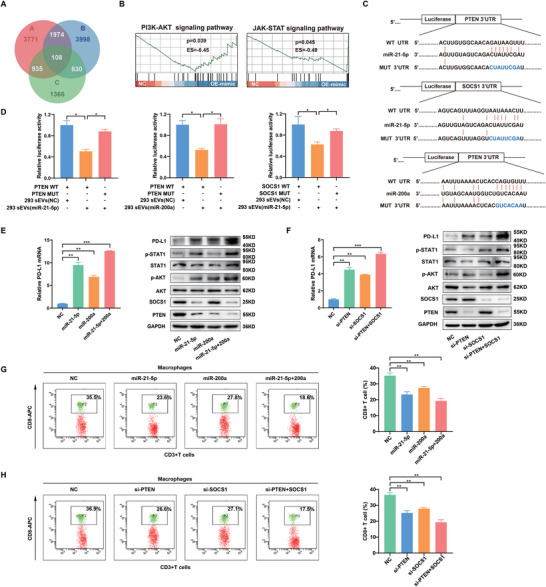
CRC‐derived sEV‐miR‐21‐5p and sEV‐miR‐200a induce PD‐L1 expression in macrophages through the PTEN/AKT and SCOC1/STAT1 pathways. A) Gene expression profiling of THP‐1‐derived macrophages transfected with NC, miR‐21‐5p, miR‐200a or miR‐21‐5p+miR‐200a was performed using RNA sequencing. Venn diagrams represent the intersections of differentially downregulated genes. B) Gene set enrichment analysis (GSEA) was used to analyze the enriched pathways of differentially downregulated genes. C) Schematic representation of the potential binding sites of miR‐21‐5p/miR‐200a in the 3′UTRs of PTEN and SOCS1. D) Luciferase assays in macrophages. A luciferase reporter vector carrying the wild‐type or mutant PTEN or SOCS1 3′UTR was cotransfected with 293T‐sEV‐miR‐21‐5p/293T sEV‐miR‐200a into macrophages, and luciferase activity was determined by dual‐luciferase reporter assay. E) PD‐L1 mRNA expression was detected in THP‐1‐derived macrophages transfected with miR‐21‐5p and/or miR‐200a using qRT‐PCR, and the expression of proteins indicated was measured in these macrophages using western blotting. F) PD‐L1 mRNA expression was detected in THP‐1‐derived macrophages transfected with si‐PTEN and/or si‐SOCS1 using qRT‐PCR, and the expression of proteins indicated was measured in these macrophages using western blotting. G) THP‐1‐derived macrophages were transfected with miR‐21‐5p and/or miR‐200a, followed by coculture with human peripheral blood lymphocytes for 72 h. Flow cytometry was performed to detect CD8^+^ T cells. H) THP‐1‐derived macrophages transfected with si‐PTEN and/or si‐SOCS1 were cocultured with human peripheral blood lymphocytes for 72 h. Flow cytometry was performed to detect CD8^+^ T cells. * *p* < 0.05, ** *p* < 0.01, *** *p* < 0.001.

To search for potential targets of miR‐21‐5p and miR‐200a in macrophages, genome‐wide expression profiling was performed with macrophages transfected with miR‐21‐5p and/or miR‐200a using RNA sequencing. A total of 108 transcripts (fold change>2 or <0.5), including PD‐L1, were deregulated in all three groups (miR‐21‐5p, miR‐200a, and miR‐21‐5p+miR‐200a) (**Figure** **6**A; Figure S8A, Supporting Information). Bioinformatic analyses revealed that these 108 genes were mostly enriched in several key cancer‐related signaling pathways (Figure 6B; Figure S8B, Supporting Information). Among these pathways, the PI3K‐AKT and JAK‐STAT pathways are known to regulate PD‐L1 expression.^[^ ^17^ ^]^ Further analyses showed that PTEN in the PI3K‐AKT pathway and SOCS1 in the JAK‐STAT pathway were significantly downregulated in miR‐21‐5p‐ and/or miR‐200a‐transfected macrophages. Two computer‐aided algorithms, TargetScan and StarBase, also identified PTEN as a potential target of miR‐21‐5p and miR‐200a and SOCS1 as a potential target of miR‐21‐5p (Figure 6C). To confirm the regulation of PTEN and SOCS1 by sEV‐miR‐21‐5p and sEV‐miR‐200a in macrophages, luciferase reporter assays were conducted. The results showed that sEV‐miR‐21‐5p could inhibit reporter expression from recombinant plasmids containing the 3’UTRs of PTEN and SOCS1, but reporters containing mutant 3’UTRs of PTEN and SOCS1 were completely refractory to sEV‐miR‐21‐5p–mediated luciferase repression in macrophages. Likewise, sEV‐miR‐200a inhibited the expression of luciferase from the recombinant plasmid containing the 3’UTR of PTEN, whereas the mutant PTEN 3’UTR was completely refractory to sEV‐miR‐200a‐mediated luciferase reporter repression in macrophages (Figure 6D).

To further determine whether sEV‐miR‐21‐5p and sEV‐miR‐200a upregulate PD‐L1 expression through the PTEN/AKT and SCOC1/STAT1 pathways, we assessed the expression of key genes in the two pathways in macrophages transfected with miR‐21‐5p and/or miR‐200a. As shown in Figure 6E, miR‐21‐5p significantly inhibited the expression of PTEN and SOCS1 and increased the expression of phosphorylated AKT (p‐AKT), phosphorylated STAT1 (p‐STAT1) and PD‐L1 in macrophages. Likewise, miR‐200a significantly inhibited PTEN expression and upregulated p‐AKT and PD‐L1 in macrophages (Figure 6E; Figure S8C,D, Supporting Information). Interestingly, macrophages transfected with miR‐21‐5p or miR‐200a, exhibited a CD206^high^/HLA‐DR^low^ phenotype (Figure S8E,F, Supporting Information). Moreover, these effects were more significant in macrophages transfected with both miR‐21‐5p and miR‐200a compared with those transfected with a single miRNA, suggesting the synergistic effects of the two miRNAs on the PTEN/AKT and SCOC1/STAT1 pathways. In addition, we observed that siRNA‐mediated PTEN or SOCS1 silencing in macrophages could phenocopy the effects of sEV‐miR‐21‐5p and sEV‐miR‐200a on PD‐L1 of macrophages (Figure 6F; Figure S9A,B, Supporting Information). Interestingly, we further observed that knockdown of PTEN significantly induced CD206 expression but inhibited HLA‐DR expression. However, knockdown of SOCS1 did not show significant impact on CD206 expression but induced HLA‐DR expression in THP1‐derived macrophages. On the whole, the knockdown of both PTEN and SOCS1 induced CD206 expression and inhibited HLA‐DR expression, suggesting that PTEN may exert more strong functions in regulating macrophage differentiation than SOCS1. It is well known that a miRNA could regulate multiple targets, and one gene also could be regulated by several miRNAs. CRC‐derived sEV or sEV‐miR‐21‐5p/sEV‐miR‐200a induced M2 like polarization and PD‐L1 upregulation in TAM, reflecting this feature of miRNAs (Figure S9C,D, Supporting Information). In addition, as expected, with ectopic expression of miR‐21‐5p and miR‐200a or knockdown of PTEN and SOCS1, macrophages decreased the CD8^+^ T cell proportion (Figure 6G,H). And these pretreated macrophages inhibited the proliferation and IL2 production of CD8^+^ T cells, which could be abolished by anti‐PD‐L1 antibodies (Figure S9E,F, Supporting Information). Taken together, these results suggest that CRC‐derived sEV‐miR‐21‐5p and sEV‐miR‐200a synergistically induce PD‐L1 expression in macrophages by regulating the PTEN/AKT and SCOC1/STAT1 pathways and thus inhibit CD8^+^ T cell functions.

### 2.7 CRC‐Derived sEV‐miR‐21‐5p and sEV‐miR‐200a Promote Tumor Growth through TAM‐Induced Immune Suppression In Vivo

**Figure 7:**
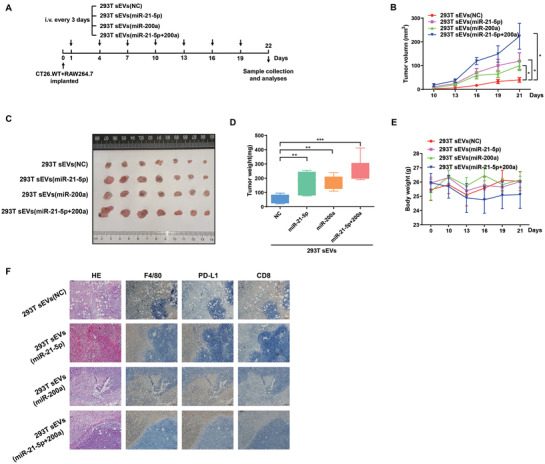
CRC‐derived sEV‐miR‐21‐5p and sEV‐miR‐200a promote tumor growth through macrophage‐induced immune suppression. A) Flow chart of the experimental design. A total of 1×10^6^ CT26.WT cells mixed with 1×10^6^ RAW264.7 cells (precultured, 1:1) were injected subcutaneously into the right flank of each BALB/c mouse. The next day, the mice were inoculated via the tail vein with 293T sEVs, 293T sEV‐miR‐21‐5p, 293T sEV–miR‐200a, or 293T sEV‐miR‐21‐5p + miR‐200a. The sEVs were administered once every 3 days (7 times, 100 µg per 100 µL per mouse) (*n* = 7 for each group). B) Tumor growth, C) tumor size, D) tumor weight, and E) body weight of the mice treated with various types of sEVs. F) HE and IHC staining were performed to detect the expression levels of F/480, PD‐L1 and CD8 in the implanted tumors. * *p* < 0.05, ** *p* < 0.01, *** *p* < 0.001.

To confirm the effects of CRC‐derived sEV‐miR‐21‐5p and sEV‐miR‐200a on tumor progression in vivo, we performed a tumor formation assay in a BALB/c mouse model. Mouse CT26.WT CRC cells mixed with mouse RAW264.7 macrophages cells (precultured, 1:1) were injected into the flank of BALB/c mice. Starting on the day after implantation, 293T sEVs (NC, miR‐21‐5p, miR‐200a, or miR‐21‐5p+miR‐200a) were injected into the mice via the tail vein every 3 days (**Figure** **7**A). sEV‐miR‐21‐5p or sEV‐miR‐200a alone significantly promoted tumor growth, and greater growth promoting effect was seen with their combination (Figure 7B–D). Mouse body weight was not significantly different among the different groups, suggesting that the sEVs were nontoxic (Figure 7E). IHC staining of mouse xenograft tumors confirmed the inverse correlation between PD‐L1^+^ macrophages and CD8^+^ T cells (Figure 7F), indicating sEV‐miR‐21‐5p and sEV‐miR‐200a treatment significantly decreased the infiltration of CD8^+^ T cells into tumor tissues. Although the total case number of mice in the four groups (*n* = 28) may be not solid enough to support the conclusion of correlation observed in clinical cohorts, the negative association between the protein levels of PD‐L1 and CD8 can be clearly observed in these xenografts (Figure S10A,B, Supporting Information). Taken together, these data show that CRC‐derived sEV‐miR‐21‐5p and sEV‐miR‐200a induce the expression of PD‐L1 in TAMs to suppress CD8^+^ T cell activities, which contributes to immunosuppression in the TME and thus promotes tumor growth in CRC.

## 3 Discussion

Functional and phenotypic plasticity is a typical feature of macrophages, which endows TAMs with complexity when exposed to different signaling stimulations in the TME.^[^ ^7^, ^18^ ^]^ In this study, we identified a novel PD‐L1^+^ CD206^+^ TAM subgroup that predicts a poor prognosis in CRC. By a series of functional and mechanistic investigations, we uncovered that CRC cells release miR‐21‐5p and miR‐200a‐enriched sEVs that strongly induce PD‐L1 expression on TAMs by regulating the PTEN/AKT and SOCS1/STAT1 pathways, promoting TAM‐mediated inhibition of CD8^+^ T cells. Thus, our data demonstrate that CRC cells can induce M2 like polarization and upregulate the PD‐L1 expression in PD‐L1^+^ CD206^+^ TAM by secreting specific sEV‐miRNAs, promoting tumor immune escape and progression.

In many cancer types, high infiltration of macrophages is associated with a poor prognosis. However, contradictory phenomena are often observed in CRC, and TAMs have been reported to be either negatively^[^ ^19^ ^]^ or positively^[^ ^20^ ^]^ associated with patient survival, with the outcome being dependent on the TME.^[^ ^7^ ^]^ Here we found that with increasing infiltration depth, TAMs exhibited increasing expression of the M2 like marker CD206. Further analyses showed that some signaling pathways related to immunosuppression were significantly enriched in TAMs, suggesting that CD206^+^ TAMs might influence CRC progression mainly through immunosuppression. Interestingly, we did not observe obvious effect of PD‐L1^+^ macrophage (PD‐L1^+^CD68^+^) on the survival in CRC; however, PD‐L1 expression was significantly correlated with M2 like macrophage levels but not M1 macrophage levels in CRC tissues, suggesting the potential important role of PD‐L1^+^CD206^+^CD68^+^TAM subgroup in CRC.

TAMs can contribute to the suppression of effective adaptive immunity by producing immunosuppressive cytokines (IL‐10 and TGF‐*β*) and metabolites (IDO and PGE2). They also express PD‐L1 and PD‐L2, which trigger checkpoint‐mediated inhibition in T cells.^[^ ^21^ ^]^ Compared with some other cancers, CRC is less responsive to anti‐PD‐1/PD‐L1 therapies and features a more macrophage‐dense TME. Therefore, macrophages are considered to be potential targets in cancer therapy. Interestingly, Pollari et al. reported that PD‐L1^+^CD68^+^ macrophages predict poor survival in patients with primary testicular lymphoma.^[^ ^22^ ^]^ However, Liu et al. reported that high PD‐L1 expression levels in CD68^+^ TAMs were correlated with better clinical outcomes.^[^ ^23^ ^]^ These conflicting data suggest the complexity of the TME in different cancer types as well as the functional variety of different TAM subgroups.^[^ ^24^ ^]^ In this study, we observed that although PD‐L1 levels are very low in CRC cells, this immune inhibitory molecule is more abundant in CD206^+^ TAMs. CD206 or PD‐L1 expression alone is not associated with CRC prognosis, but the abundance of PD‐L1^+^CD206^+^ TAMs is significantly associated with a poor prognosis in CRC.

PD‐L1 is a glycoprotein that induces T cell anergy and apoptosis by activating the receptor PD‐1. Here we observed a negative correlation between PD‐L1^+^CD206^+^ macrophages and CD8^+^ T cells in CRC tissues. Further investigations based on single‐cell sequencing data and co‐culture assays confirmed that CRC cells upregulate PD‐L1 expression in TAMs and that CRC‐educated macrophages inhibit the proliferation and function of CD8^+^ T cells. These data demonstrate that CRC‐induced PD‐L1 expression in TAMs may mediate immune escape. Therefore, it is reasonable to conclude that blocking PD‐L1 signaling in TAMs appears to be a promising choice for CRC treatment and reverses immune suppression in tumors by reinvigorating CD8^+^ T cells; this approach may also be an alternative choice to augment the efficiency of anti‐PD‐L1 treatment in CRC.^[^ ^25^ ^]^

PD‐L1 expression has been identified in multiple cell types, including various cancer cells and immune cells.^[^ ^26^ ^]^ As a critical molecule affecting the efficacy of ICB treatment, PD‐L1 undergoes complicated regulation that has been intensively investigated. Some factors, including STAT3, HIF‐1a, NF‐Kb, and CDK4/CDK6, are known to be involved in the regulation of PD‐L1 expression.^[^ ^17^, ^27^ ^]^ Prima et al. demonstrated that the COX2/mPGES1/PGE2 pathway regulates PD‐L1 expression in TAMs and myeloid‐derived suppressor cells.^[^ ^28^ ^]^ However, how PD‐L1 expression is upregulated in CRC TAMs is unclear.

sEVs are secreted by almost all cell types and exert intercellular communication and cargo transfer functions, and miRNAs appear to be a group of the key signaling molecules in sEVs. Recent studies have shown that sEV‐miRNAs are emerging as key regulatory molecules mediating intercellular communication in the TME.^[^ ^29^ ^]^ MiRNAs in cancer cell‐derived sEVs were shown to aid tumor immune evasion and could be therapeutic targets in many cancer types.^[^ ^30^ ^]^ We previously demonstrated that tumor‐secreted miR‐214 induces regulatory T cells and promotes immune evasion and tumor growth^[^ ^12^ ^]^ and showed that sEV‐miRNAs appear to be promising tumor biomarkers and therapeutic agents.^[^ ^9^, ^31^ ^]^ Recent studies by others^[^ ^32^ ^]^ have also suggested that CRC cells can regulate other cells and promote tumor progression^[^ ^33^ ^]^ by secreting sEV‐miRNAs.^[^ ^34^ ^]^ Here, we investigated how PD‐L1 expression is enhanced in CRC TAMs. According to miRNA‐sequencing data for CRC cell‐derived sEVs and subsequent qRT‐PCR validation in plasma sEVs from CRC patients, four miRNAs (miR‐21‐5p, miR‐1246, miR‐200a, and miR‐92a‐3p) were found to be most enriched in CRC sEVs. Further studies revealed that CRC‐secreted sEV‐miR‐21‐5p and sEV‐miR‐200a could synergistically regulate PD‐L1 expression in TAMs.

A recent work by Wang and colleagues showed that exosomal miRNAs (miR‐25‐3p, miR‐130b‐3p, and miR‐425‐5p) derived from CXCR4 overexpressing CRC cells could induce macrophage M2 like polarization which, in turn, promoted cancer metastasis.^[^ ^35^ ^]^ CRC‐derived exosomal miR‑934 can also induce M2 like polarization in macrophages by inhibiting PTEN expression,^[^ ^34^ ^]^ and exosomal miR‐1246 from p53‐mutant cancers promotes immunosuppression by reprogramming macrophages.^[^ ^36^ ^]^ In addition, endoplasmic reticulum‐stressed liver cancer cells can secrete exosomal miR‐23a‐3p and induce PD‐L1 expression by regulating the PTEN‐AKT axis in macrophages.^[^ ^37^ ^]^ We also revealed that CRC‐derived sEV‐miR‐21‐5p and sEV‐miR‐200a could induce macrophage PD‐L1 expression by inhibiting PTEN. From these data, we concluded that sEV‐miRNAs are key signaling molecules that regulate macrophage M2 like polarization and PD‐L1 expression in TAMs and that the tumor suppressor PTEN is a key target mediating these remote regulatory effects on macrophages by cancer cells. In addition, JAK‐STAT signaling pathway could upregulate PD‐L1 expression, and targeting STAT1/3 has been suggested as an alternative or sensitizing agent of PD‐L1 blockage therapy. SOCS1 is a key negative regulatory factor in JAK/STAT1 pathway. We showed that CRC‐derived sEV‐miR‐21‐5p activate STAT1 signaling by directly targeting SOCS1. These data demonstrate that miR‐21‐5p and miR‐200a, two well‐known oncogenic miRNAs, also could exert tumor‐promoting functions by mediating intercellular “crosstalk” between cancer cells and TAMs.

In conclusion, this study revealed a specific PD‐L1^+^CD206^+^ macrophage subgroup, which was induced by CRC‐derived multiple sEV‐miRNAs and predict a poor prognosis in CRC. This TAM subgroup promotes tumor growth by inhibiting CD8^+^ T cell activity and thus inducing an immunosuppressive TME. Hence, inhibiting the secretion of specific miRNAs from CRC and targeting PD‐L1 in TAMs may serve as novel methods for CRC treatment and a setization method for anti‐PD‐L1 therapy in CRC.

## 4 Experimental Section

### Cell Culture and Treatments

The human CRC cell line SW620, human normal colonic epithelial cell line NCM460, murine colon cancer cell line CT26, human monocytic leukemia cell line THP‐1, murine macrophage line RAW264.7, and HEK‐293T (293T) cells were obtained from ATCC and cultured following the provider's instructions except that EV‐free FBS was added in cell culture media. EV‐free FBS was obtained by ultracentrifuging overnight (110 000g, 4 ℃). Cells were confirmed to be free of mycoplasma and were characterized by Genewiz Inc. (China) using short tandem repeat markers. THP‐1 monocytes were differentiated into macrophages by incubation with 100 × 10^−9^ m phorbol 12‐myristate 13‐acetate (PMA) for 24 h.

For coculture experiments, macrophages were seeded in the lower chamber in a 6‐well plate, and CRC cells were added to the upper chamber of a Transwell insert with a 0.4‐µm pore. In some experiments, the abovementioned macrophages were further coincubated with lymphocytes isolated from the peripheral blood of healthy donors. In other experiments, sEVs (10 µg per 10^5^ cells) extracted from the supernatants of different cells were cocultured with macrophages. After coincubation for 48 h, macrophages were collected for flow cytometry, western blot or quantitative reverse transcription‐polymerase chain reaction (qRT‐PCR) analyses, and the supernatants were used for cytokine measurements.

### Patients and Tissue Samples

Tumor tissue or blood samples were obtained from CRC patients who underwent surgery at Affiliated Hospital of Jiangnan University. All clinicopathological diagnoses were confirmed by at least two pathologists according to the guidelines of the American Joint Committee on Cancer (AJCC). The present study was approved by the Ethics Committee of Affiliated Hospital of Jiangnan University (LS2018022), and informed consent was obtained from all patients before enrollment.

### Isolation and Analyses of sEVs

sEVs were isolated from cell culture medium or plasma through differential centrifugation as previously described.^[^ ^9^ ^]^ Plasma samples were diluted in PBS before centrifugation. Briefly, after removal of cells and other debris by centrifugation at 300 g for 10 min, 2000 g for 10 min, 10000 g for 30 min, cell supernatant was centrifuged at 110 000 × *g* for 70 min (all of these steps were performed at 4°C). The sEVs were collected and resuspended in PBS or FBS‐free medium. A NanoSight N300 instrument (Malvern, UK) was used to analyze the distribution of sEVs sizes. sEVs quantification was conducted by measuring the protein content of sEVs pellets with the Pierce BCA Protein Assay Kit (CWBio, China).

### RNA Sequencing

MiRNA mimics (miR‐21‐5p or miR‐200a) were transfected into macrophages using Lipofectamine 2000 (Invitrogen). Forty‐eight hours after transfection, the macrophages were collected and subjected to RNA sequencing.

### Luciferase Reporter Assays

Reporter plasmids containing wild‐type (WT) or mutant (MUT) 3’UTRs of PTEN or SOCS1 (psiCHECK2‐PTEN‐WT, psiCHECK2‐PTEN‐MUTt, psiCHECK2‐SOCS1‐WT, and psiCHECK2‐SOCS1‐MUT) were constructed and transfected into macrophages. Then, the macrophages were coincubated with miR‐21‐5p‐ and/or miR‐200a‐overexpressing sEVs. Three days after transfection, reporter gene activity was measured with a dual‐luciferase reporter assay kit (Beyotime, China) according to the manufacturer's instructions.

### Analyses of the miRNA Profiles of sEVs by Next‐Generation Sequencing

Total RNA was extracted from sEVs using TRIzol reagent (Invitrogen) according to the manufacturer's instructions. MiRNA expression profiles were determined by RNA‐seq at RiboBio (China) using a HiSeq3000 (Illumina).

### Western Blotting

Cells or sEVs were lysed in RIPA buffer, and proteins were collected and denatured. The proteins were subjected to Western blotting analyses as previously described.^[^ ^4^ ^]^ The detailed information of antibodies used was listed in Table S1 in the Supporting Information.

### Flow Cytometry

Macrophages were stained with fluorochrome‐conjugated monoclonal antibodies against HLA‐DR (eBioscience) and CD206 (eBioscience) according to the manufacturer's instructions. Peripheral blood lymphocytes (PBLs) were extracted from the peripheral blood of healthy donors with a lymphocyte isolation reagent. PBLs were cocultured with macrophages or sEVs that were pretreated in different ways and stained with PE‐conjugated anti‐CD3 or APC‐conjugated anti‐CD8a antibodies (eBioscience). Staining of cells with IgG isotype were considered as negative control. The cells were then subjected to flow cytometric analyses on a BD FACS CantoII or Agilent BIO ACEA NovoCyte flow cytometer and analyzed using FlowJo software (TreeStar).

### Tumor Formation in Mice

CT26.WT cells and RAW264.7 cells were mixed and then subcutaneously injected into the right flank of 6‐week‐old male BALB/c mice. sEVs were injected into the mice via the tail vein at the same dose (100 µg per 100 µL) every 3 days. The mice were sacrificed after 7 injections for data collection. All animal experimental procedures were approved by the Ethical Committee of Affiliated Hospital of Jiangnan University (JN.No2020915b0321115).

### Immunofluorescence (IF) Staining and Multiplex Immunofluorescence (mIF) Staining

PKH67‐labeled sEVs were cocultured with macrophages at 37°C for 24 h. Then, the macrophages were fixed with 4% paraformaldehyde, permeabilized with 0.01% Triton‐100 for 10 min, and counterstained with DAPI for nuclear staining. For in vivo experiments, PKH67‐labeled sEVs were injected into mice via the tail vein at the same dose (100 µg per 100 µL) every other day. The mice were sacrificed after a period of 7 days, and then mouse peritoneal macrophages were extracted and further plated in 12‐well plates. The following experimental steps were the same as those in the in vitro IF assay described above. All images were captured on an Olympus fluorescence microscope equipped with visualization software. For mIF assays, primary antibodies against CD206 (1:200, Proteintech), CD8 (1:200, eBioscience) and PD‐L1 (1:50, Cell Signaling Technology) were applied. A PANO 7‐plex IHC kit (Panovue, China) was used for immunofluorescence staining, and images were obtained with the Mantra System (PerkinElmer, USA).

### Pseudotime Trajectory and Immune Signature Gene Expression Analyses

The expression matrix and clinical information for the TCGA COAD‐READ dataset, which contains 582 tumor samples and 51 normal samples, was obtained from University of California Santa Cruz (UCSC) Xena. Single cell RNA sequencing(scRNA‐seq)data and metadata information are available in the NCBI Gene Expression Omnibus (GEO) databases (GSE132465, GSE132257 and GSE144753). Two additional microarray expression datasets (GSE39582 and GSE87211) were downloaded from GEO to increase the reliability of the analyses. These two datasets contain 566 and 203 tumor samples, respectively. The ssGSEA algorithm^[^ ^38^ ^]^ was used to evaluate the infiltration abundance of 28 kinds of immune cells in TCGA COAD‐READ data set, and the CD8^+^ T cells and macrophage infiltration score of each patient were extracted. These cases with both TAM infiltration and positive expression of CD206/PD‐L1 were subjected to the correlation analyses to CD8^+^ T cells.

To reveal phenotypic changes in macrophages during the tumor‐mediated education process, Monocle 2, an R package designed for single‐cell trajectories, was used. Trajectories were visualized as 2D tSNE plots, while dynamic expression heatmaps were constructed using the plot‐pseudotime heatmap function.

The mean normalized expression levels for each cell cluster determined by the immune signature gene expression analysis were calculated and then normalized into row Z scores to represent the relative expression levels among different cell clusters. These data are presented in the form of a box plot.

### Statistical Analyses

SPSS 16.0 software (SPSS Inc.) was used for statistical analyses. Experimental results are shown as the mean ± SD. A two‐tailed Student's *t* test or the Wilcoxon‐Mann‐Whitney test was performed to evaluate differences between two sets, and one‐way analysis of variance (ANOVA) was employed to compare three or more sets. Kaplan‐Meier survival analysis and the Cox proportional hazards model were used to analyze the association between the different groups prognosis with the R package “Survminer”. Correlation analyses were performed using the Pearson correlation method; a *p* value <0.05 and *r* >0.4 were considered statistically significant. *p* values ≤ 0.05 were considered to be statistically significant.

### Ethical Approval and Consent to Participate

The present study was approved by the Institutional Ethical Committee of the affiliated hospital of Jiangnan University.

### Consent for Publication

We have received consents from individual patients who have participated in this study. The consent forms will be provided upon request.

## Conflict of Interest

The authors declare no conflict of interest.

## Author Contributions

Y.Y., SL.H., and ZH.H. developed study concepts and design; Y.Y., BX.L., YL.C., SR.Y., YH.L., GY.J., Y.Q., and Y.C. performed experiments, analyzed, and interpreted data. BX.L., KS.C., and SL.H. performed bioinormatic analyses; Y.Y. and B.L. performed statistical analyses; LY.Z., ZH.B., and BJ.F. managed patients and collected clinical samples; Y.Y., B.L., and Z.H. obtained funding. Y.Y., BX.L., SL.H., and Z.H. wrote the manuscript; ZH.H. provided study supervision.

## Supporting information

Supporting Information

Supporting Information

Supporting Information

Supporting Information

Supporting Information

## Data Availability

All the data supporting the conclusions of this article are included within the article and the supplementary files.
