# Supplementary material for: Colorectal Cancer‐Derived Small Extracellular Vesicles Promote Tumor Immune Evasion by Upregulating PD‐L1 Expression in Tumor‐Associated Macrophages
Source: Adv Sci (Weinh). 2022 Jan 17;9(9):2102620. doi: 10.1002/advs.202102620 (PMC8948581; doi:10.1002/advs.202102620)
Supplement: Supplementary file 1 — Supporting Information [file ADVS-9-2102620-s005.pdf]

ADVANCED SCIENCE
Open Access

# Supporting Information

for *Adv. Sci.*, DOI 10.1002/advs.202102620

Colorectal Cancer-Derived Small Extracellular Vesicles Promote Tumor Immune Evasion by Upregulating PD-L1 Expression in Tumor-Associated Macrophages

*Yuan Yin, Bingxin Liu, Yulin Cao, Surui Yao, Yuhang Liu, Guoying Jin, Yan Qin, Ying Chen, Kaisa Cui, Leyuan Zhou, Zehua Bian, Bojian Fei, Shenglin Huang* and Zhaohui Huang**

ADVANCED SCIENCE
Open Access

# Supporting Information

for *Adv. Sci.,* DOI: 10.1002/advs.202102620

# Colorectal Cancer-Derived Small Extracellular Vesicles Promote Tumor Immune Evasion by Upregulating PD-L1 Expression in Tumor-Associated Macrophages

*Yuan Yin, Bingxin Liu, Yulin Cao, Surui Yao, Yuhang Liu, Guoying Jin, Yan Qin, Ying Chen, Kaisa Cui, Leyuan Zhou, Zehua Bian, Bojian Fei, Shenglin Huang,* and Zhaohui Huang**

**Supplementary Materials for**

# Colorectal cancer-derived small extracellular vesicles promote tumor immune evasion by upregulating PD-L1 expression in tumor-associated macrophages

**Authors:**

Yuan Yin[1,2,#], Bingxin Liu[1,2,#], Yulin Cao[1,2], Surui Yao[1,2], Yuhang Liu[1,2], Guoying Jin[1,2], Yan Qin[3], Ying Chen[1,2], Kaisa Cui[1,2], Leyuan Zhou[4], Zehua Bian[1,2], Bojian Fei[5], Shenglin Huang[6,*], Zhaohui Huang[1,2,*]

**Affiliations:**

Dr. Y. Yin, Mr. B. Liu, Ms. Y. Cao, Ms. S. Yao, Mr. Y. Liu, Ms. G. Jin, Ms. Y. Chen, Dr. K. Cui, Dr. Z. Bian and Prof. Z. Huang

Wuxi Cancer Institute, Affiliated Hospital of Jiangnan University, Wuxi 214062, China;

Laboratory of Cancer Epigenetics, Wuxi School of Medicine, Jiangnan University, Wuxi 214122, China

Ms. Y. Qin

Department of Pathology, Affiliated Hospital of Jiangnan University, Wuxi, Jiangsu, China.

Dr. L. Zhou

Department of Radiation Oncology, Affiliated Hospital of Jiangnan University, Wuxi 214062, China

Dr. B. Fei

Department of Gastrointestinal Surgery, Affiliated Hospital of Jiangnan University, Wuxi 214062, China

Prof. S. Huang

Fudan University Shanghai Cancer Center and Institutes of Biomedical Sciences,

Fudan University, Shanghai 200032, China
[#] These authors contributed equally to this work.

***Correspondence:**

**Zhaohui Huang,** Wuxi Cancer Institute, Affiliated Hospital of Jiangnan University, Wuxi 214062, China. Tel/Fax: +86-510-88682087. Email: zhaohuihuang@jiangnan.edu.cn.

**Shenglin Huang,** Fudan University Shanghai Cancer Center and Institutes of Biomedical Sciences, Fudan University, Shanghai 200032, China. Tel/Fax: +86-21-34777597. Email: slhuang@fudan.edu.cn.

## Supplementary Materials and Methods

### Construction of stable cell lines

PWPXL-NC, PWPXL-miR-21, or PWPXL-miR-200a plasmids were transfected into HEK-293T cells along with the packaging plasmid ps-PAX2 and the envelope plasmid pMD2G using Lipofectamine 2000 (Invitrogen, USA). Virus particles were harvested 48 h after cotransfection and then individually used to infect HEK-293T cells to generate corresponding stable cell lines. The efficiency of miR-21-5p or miR-200a overexpression was assessed using qRT-PCR.

### RNA sequencing and bioinformatic analyses

MiRNA mimics were transfected into macrophages using Lipofectamine 2000 (Invitrogen). Forty-eight hours after transfection, the macrophages were collected and subjected to RNA sequencing. The DESeq2 package of R software was used to normalize the expression data and analyze differentially expressed genes (fold change>2 or <0.5, and adjusted p-value<0.05). Genes downregulated by miR-21-5p and/or miR-200 mimics were subjected to gene enrichment analyses using Gene Set Enrichment Analysis (GSEA). The online miRNA prediction tools TargetScan and miRTarBase were used to comprehensively predict the targets of miR-21-5p or miR-200a.

**RNA isolation and qRT-PCR**

Total RNA was extracted from cells or sEVs using RNAiso Plus (TaKaRa) and then reverse transcribed into cDNA using the PrimeScript II 1st Strand Synthesis Kit (TaKaRa). qRT-PCR was performed on a ViiA7 real-time PCR system using UltraSYBR Mixture (CWBio). Relative gene expression levels were normalized to those of *ACTB* and calculated utilizing the $2^{-\Delta\Delta t}$ method.

**Immunohistochemistry (IHC)**

IHC was performed on 4-μm sections of paraffin-embedded tissue samples to detect the protein expression levels of CD68, CD206, CD3, CD8, and PD-L1. In short, sections were deparaffinized, rehydrated and immersed in 10 mM citrate buffer for heat-induced antigen recovery. Next, the slides were stained using antibodies against CD68 (1:200, Santa Cruz, USA), CD206 (1:200, Proteintech, USA), CD3 (1:200, eBioscience, USA), CD8 (1:200, eBioscience) and PD-L1 (1:50, Cell Signaling Technology, USA) at 4°C overnight. The subsequent steps were performed using the EnVision FLEX High pH 9.0 Visualization System (DAKO, Denmark). All slides were independently evaluated by two pathologists. The staining intensity was visually scored and stratified (score 0-3) as previously described: negative staining, 0 (-); weakly stained, 1 (+); moderately stained, 2 (++); and strongly stained, 3 (+++).

**Identification of PD-L1+ CD206+ TAM subgroup**

Firstly, for single cell sequencing data, according to the cell annotation information uploaded by Lee et al., the gene expression data of myeloid cells are extracted from the expression profile matrix. Subclustering was performed for the four major cell types defined in the initial clustering using the graph-based algorithm in Seurat. For each cluster subset, differentially expressed genes were selected with a mean expression between 0.0125 and 3 and a dispersion of more than 0.5 using the FindVariableGenes function. According to the differentially expressed genes among these different clusters of macrophages, corresponding molecular characteristic maps were drawn. Principal component analyses were performed using these genes; the number of principal components was differentially selected from the knee point of the scree plot for each cell type to accommodate different population complexities.

Resolutions from 0.2 to 1.6 were explored for better subcluster representation.

Secondly, for bulk RNA sequencing data from TCGA and GEO datasets, the CIBERSORT algorithm was applied to quantify the absolute amount of 22 infiltrating immune cell types in each sample. CIBERSORT employs deconvolution of bulk gene expression data and a sophisticated algorithm for in silico quantification based on a leukocyte gene signature matrix, termed LM22, which contains 547 genes that distinguish 22 human hematopoietic cell phenotypes. Among the 22 infiltrating immune cell types, there are three macrophages types (M0, M1, and M2). CIBERSORT algorithm indicates the presence of M2 macrophage infiltration in tumor tissues, which will be used for further analyses. Based on the gene expression of PD-L1 and CD206 in these tumor samples, we calculated the ratio of them and grouped these tumors into corresponding high or low groups (PD-L1/CD206 ratio: SPP1/CD68 > 1, high group; PD-L1/CD206 < 1, low group).

Thirdly, for tumor tissue samples obtained from CRC patients who underwent surgery at our hospital. All IHC slides were independently evaluated by at least two pathologists. The staining intensity was visually scored and stratified (score 0-3) as previously described: negative staining, 0 (-); weakly stained, 1 (+); moderately stained, 2 (++); and strongly stained, 3 (+++). CD68 and CD206 both positive cells in CRC tissues were regarded as TAMs, and among these TAMs, PD-L1 positively staining cells were identified as PD-L1+ CD206+ TAM subgroup.

**Enzyme linked immunosorbent assay (ELISA)**

ELISA reagents (duo-set kit) for human IL-2 were purchased from ExCell Bio (China), and this assay was performed according to the manufacturer's instructions. Absorbance was measured with a wavelength correction (A450 nm) using a microplate reader.

**Generation of miRNA "sponge"**

For the miR-21-5p or miR-200a knockdown plasmid, a sequence encoding a string of specific antisense sequences to miR-21-5p or miR-200a (sponge) was designed as proposed by a previous study (Ma et al., 2011). The sponge construct contained three copies of an antisense sequence for each miRNA. Then, the sponge construct was

cloned into the same vector and co-expressed with GFP under the same promoter control.

## Supplementary Figures

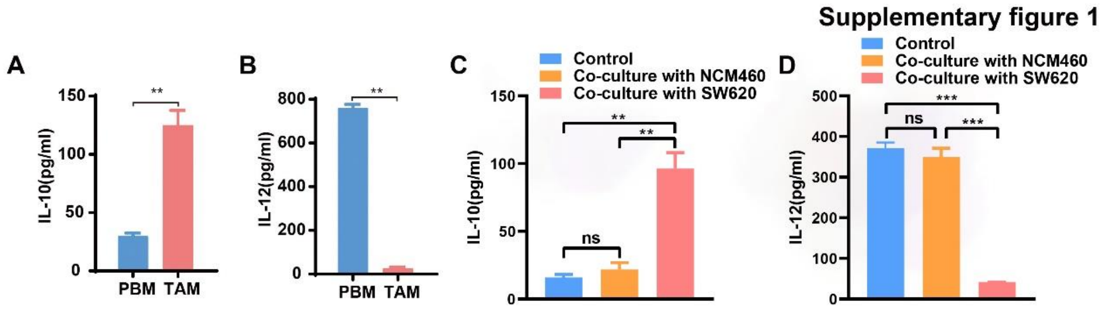


**Figure S1.** IL-10 and IL-12 levels in TAMs from CRC tissues and freshly isolated human monocytes.

A, B) IL-10 and IL-12 levels were detected in CRC TAM and PBM by ELISA.

C, D) IL-10 and IL-12 levels were detected in the culture supernatants of macrophages cocultured with SW620 or NCM460 cells by ELISA. * $P < 0.05$, ** $P < 0.01$, *** $P < 0.001$.

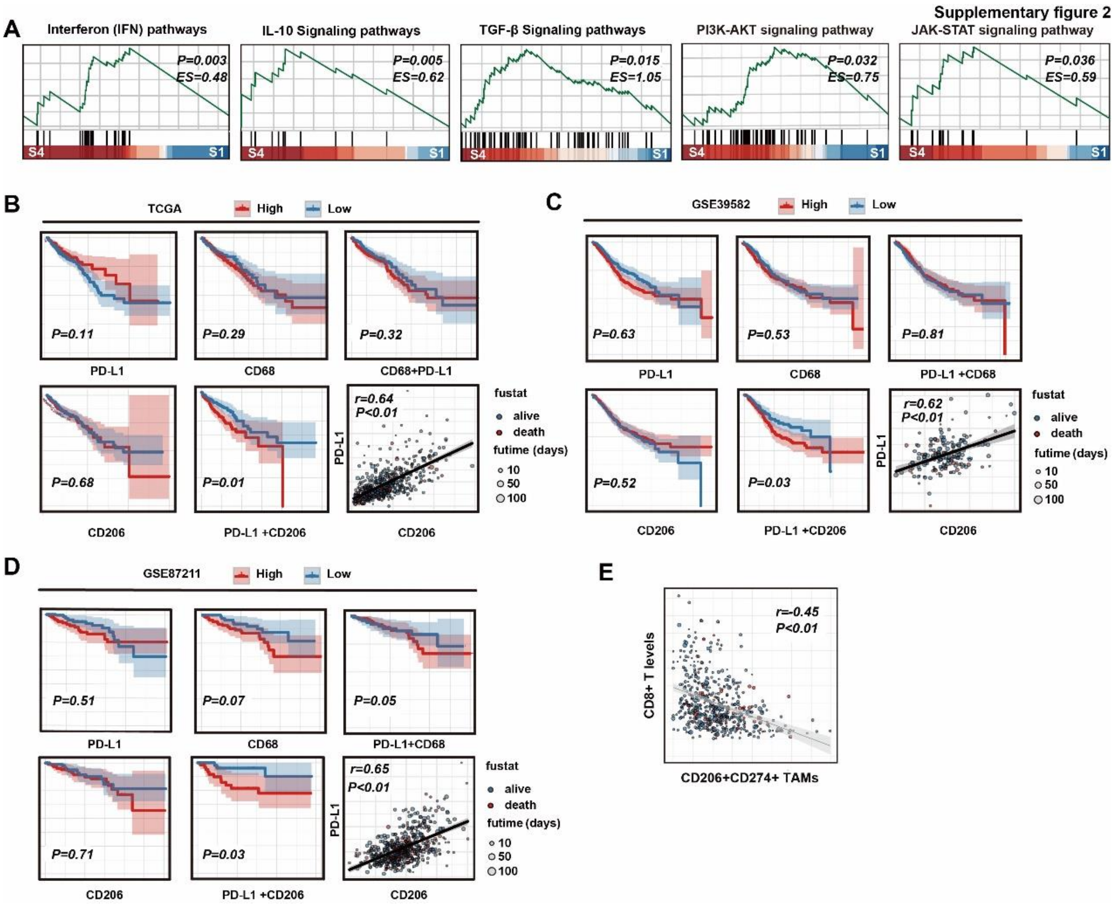


**Figure S2.** PD-L1$^{+}$CD206$^{+}$ macrophages in CRC tissues are associated with a poor prognosis.

A) GSEA analyses using the differentially expressed genes of TAMs during the tumor-mediated education process.

B) Survival analyses of CRC based on the expression levels of CD68, CD206, PD-L1, CD68 combined with PD-L1, and CD206 combined with PD-L1 in the TCGA COAD-READ dataset. Correlation analysis of PD-L1 and CD206 in CRC.

C) Survival analyses of CRC based on the expression levels of CD68, CD206, PD-L1, CD68 combined with PD-L1, and CD206 combined with PD-L1 in the GSE39582 dataset. Correlation analysis of PD-L1 and CD206 in CRC.

D) Survival analyses of CRC based on the expression levels of CD68, CD206, PD-L1, CD68 combined with PD-L1, and CD206 combined with PD-L1 in the GSE87211 dataset. Correlation analysis of PD-L1 and CD206 in CRC.

E) Correlation analysis between CD8+Tcell levels and the abundance of PD-L1$^{+}$CD206$^{+}$ TAMs in CRC tissues. * $P < 0.05$, ** $P < 0.01$, *** $P < 0.001$.

Supplementary figure 3

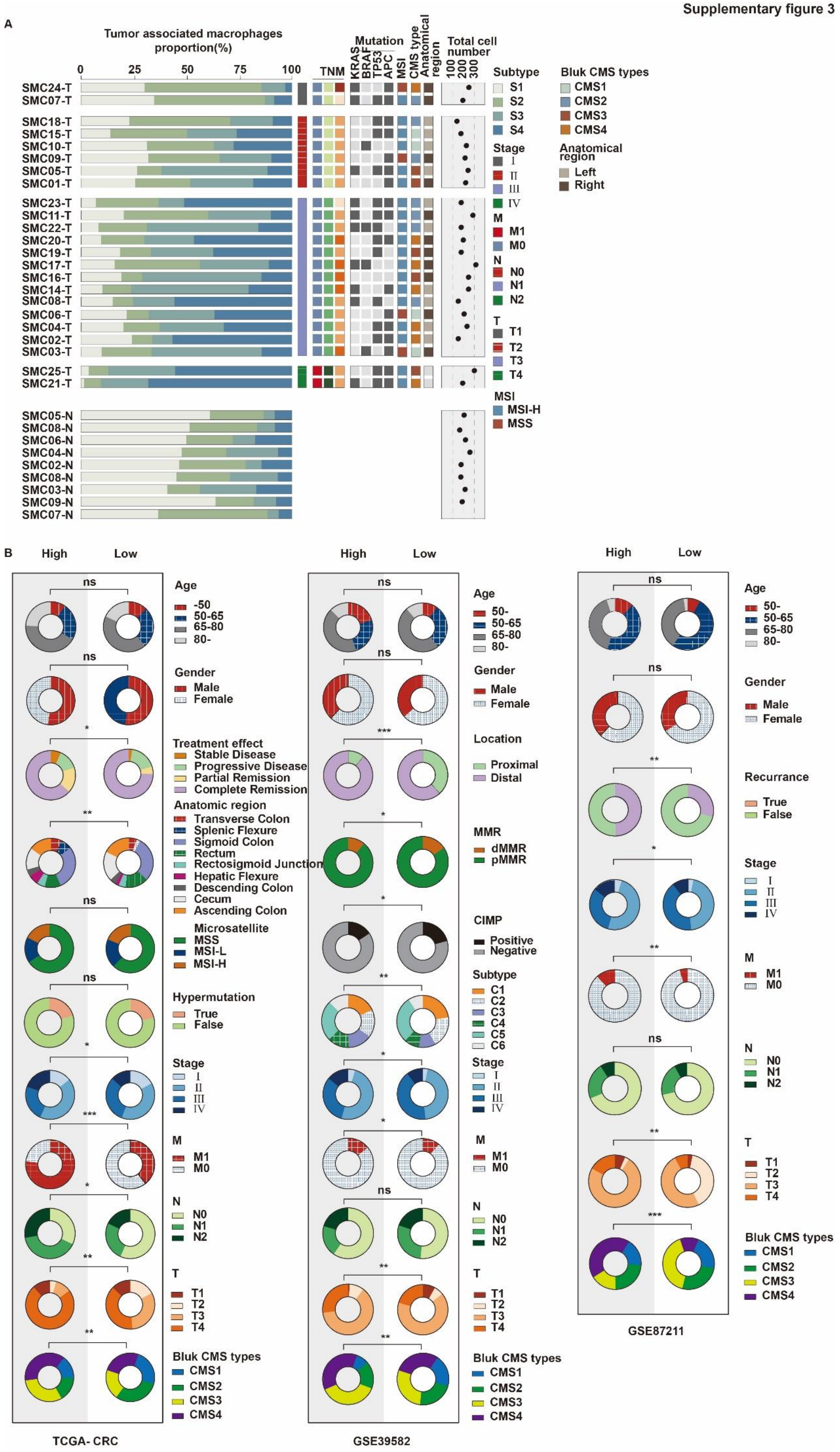

**Figure S3.** Single cell and transcriptome levels in CRC tissues from databases reveal the correlation between two subtypes of macrophages and clinicopathological indexes.

A) Cell numbers (right) and relative proportions (left) of macrophages subsets color-coded by subtype. Clinical and genetic information is aligned on the middle.

B) Pie charts showing the Chi-squared tests of clinicopathologic factors for two subtypes of macrophages (High/Low) in CRC tumor samples from the TGCA and GEO.

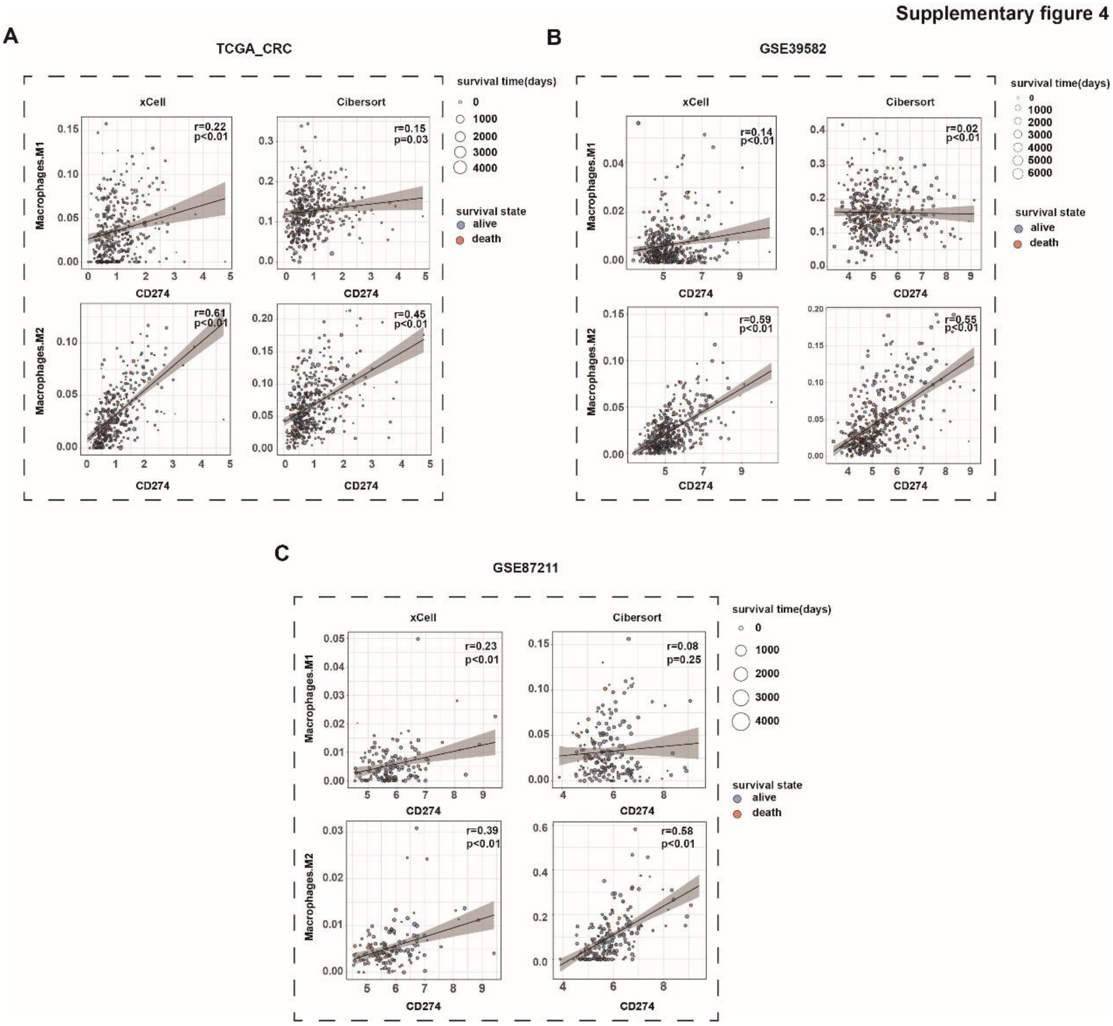


**Figure S4.** The Pearson correlation between CD274 expressions and M1 or M2 type macrophages infiltrates of CIBERSORTx and xCell in multiple public CRC datasets A) TCGA. B) GSE39582. C) GSE87211. Pearson's r and p values are shown for each plot.

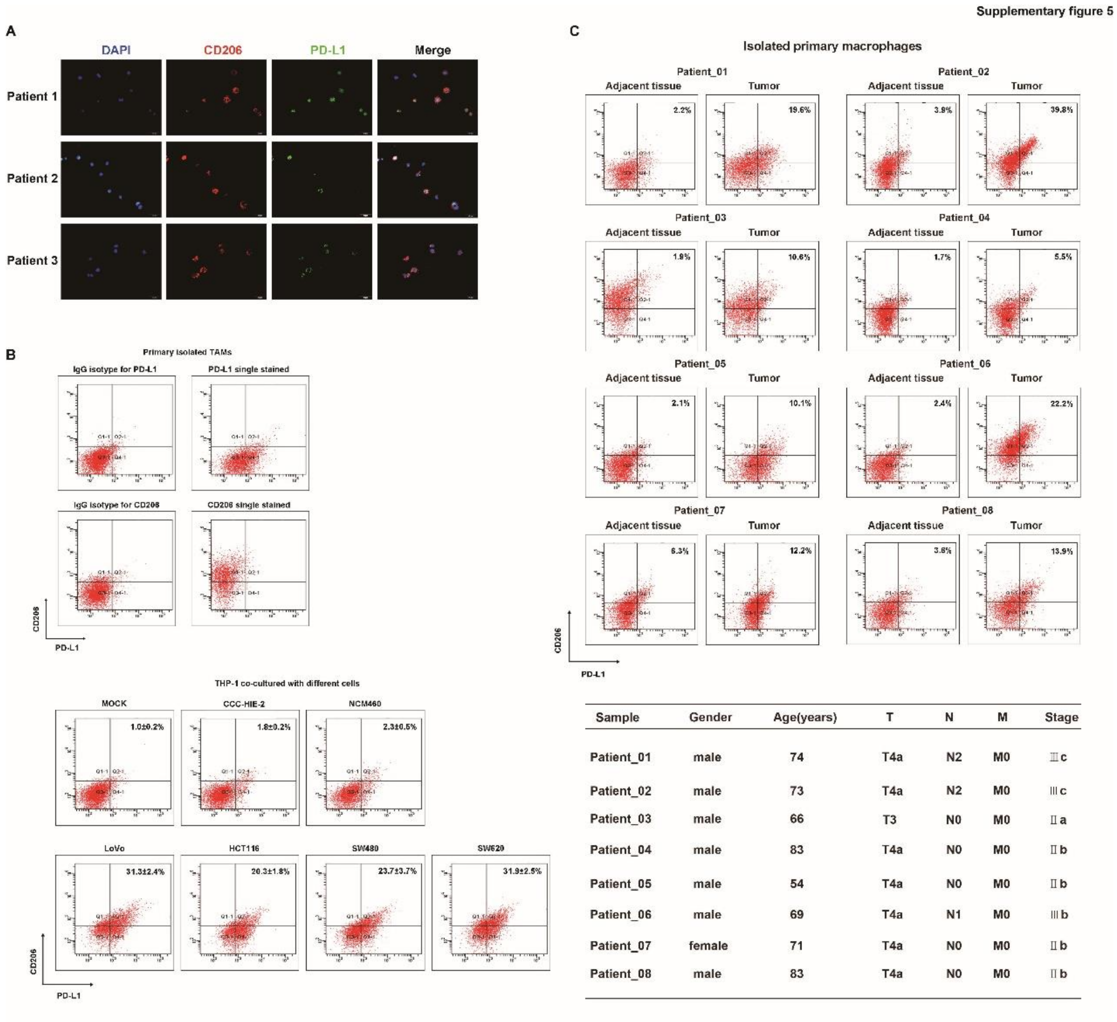


| Sample | Gender | Age(years) | T | N | M | Stage |
|---|---|---|---|---|---|---|
| Patient_01 | male | 74 | T4a | N2 | M0 | Ⅲc |
| Patient_02 | male | 73 | T4a | N2 | M0 | Ⅲc |
| Patient_03 | male | 66 | T3 | N0 | M0 | Ⅱa |
| Patient_04 | male | 83 | T4a | N0 | M0 | Ⅱb |
| Patient_05 | male | 54 | T4a | N0 | M0 | Ⅱb |
| Patient_06 | male | 69 | T4a | N1 | M0 | Ⅲb |
| Patient_07 | female | 71 | T4a | N0 | M0 | Ⅱb |
| Patient_08 | male | 83 | T4a | N0 | M0 | Ⅱb |

**Figure S5. Correlation analyses of CD206 and PD-L1 in macrophages.**

A) Primary macrophages were isolated from fresh CRC tissues and subjected to the detection CD206 and PD-L1 by immunofluorescence.

B) Double-stained flow cytometry analyses of CD206 and PD-L1 for the macrophages cocultured with CRC cells. Primary CRC TAMs were isolated to stain CD206 or PD-L1, respectively, to help to clearly define the corresponding “positive” or “negative” cells as well as to designate quadrant gates. Staining of cells with IgG isotypes were set as negative controls.

C) A total of 8 fresh CRC and their adjacent tissues were collected, and primary macrophages were isolated to detect the expression of CD206 and PD-L1 by the double-stained flow cytometry.

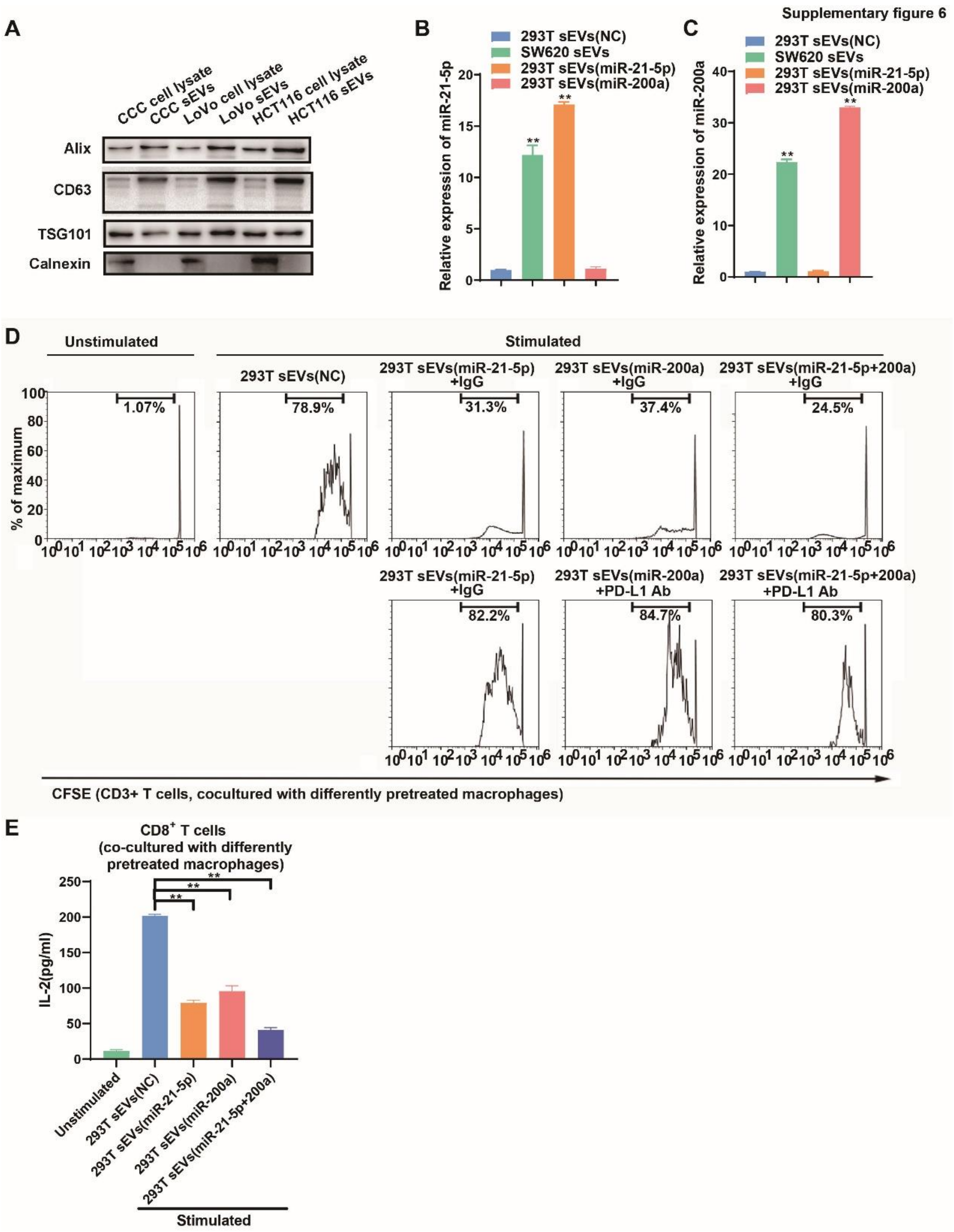


**Figure S6.** sEV-miR-21-5p and sEV-miR-200a promote the proliferation and function of CD8$^+$ T cells.

A) Western blot analyses were performed to detect typical sEVs biomarkers (CD63, TSG101 and Alix as positive markers, Calnexin as negative marker) in sEVs derived from CRC cell lines or normal colonic epithelial cells and the corresponding cells.

B, C) qRT-PCR was used to detect the levels of miR-21-5p (B) or miR-200a (C) in

sEVs derived from 293T, SW620, 293T cells stably overexpressing miR-21-5p or miR-200a.

D, E) Representative histogram of CFSE-labeled human peripheral $CD8^+$ T cells cocultured with differently pretreated macrophages. THP-1-derived macrophages were treated with 293T sEV-miR-21-5p and/or 293T sEV-miR-200a as well as anti-PD-L1 antibodies (10ug/ml) (D). IL-2 levels in these $CD8^+$ T cells were detected by ELISA (E). * $P < 0.05$, ** $P < 0.01$, *** $P < 0.001$.

Supplementary figure 7

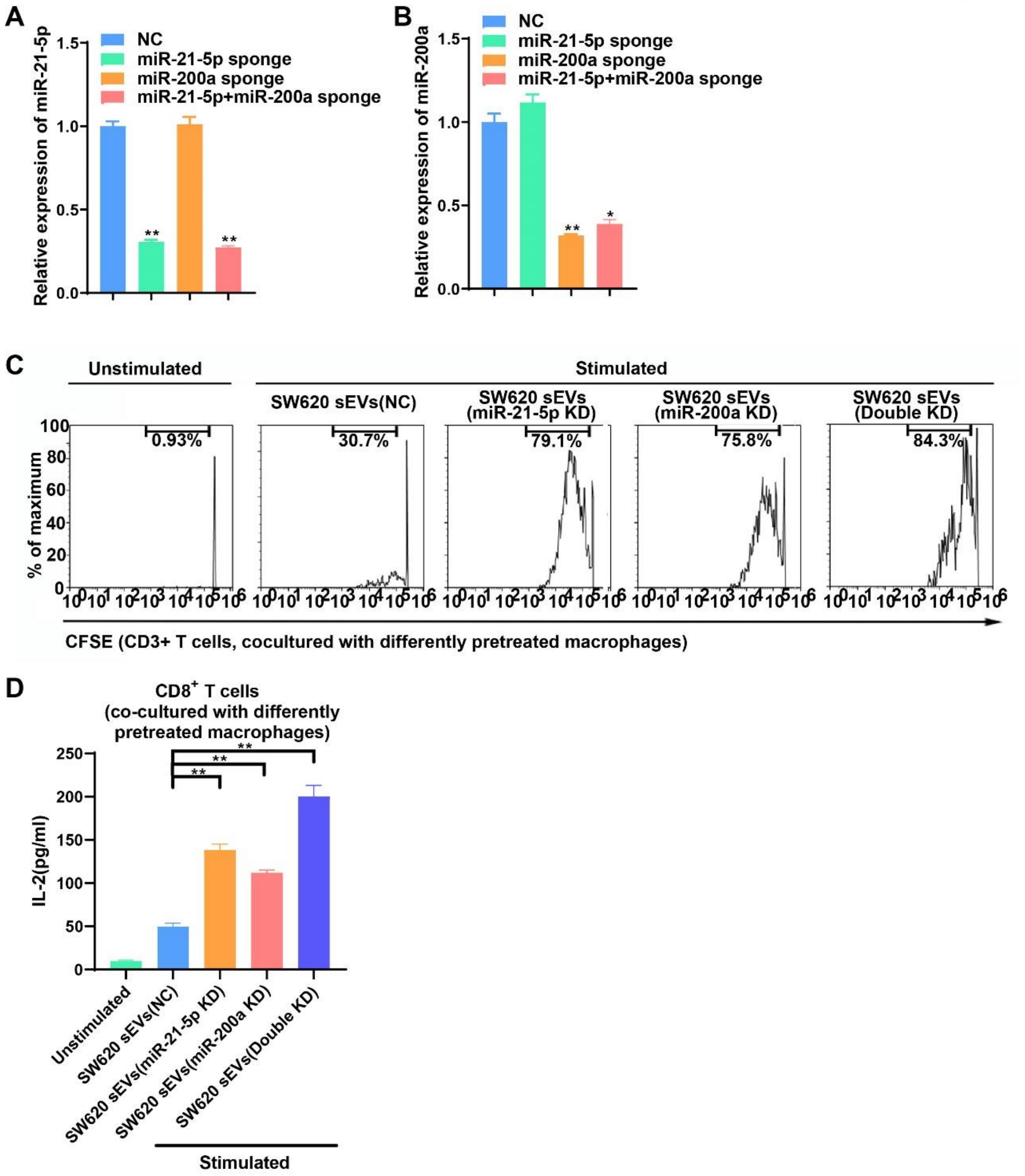


**Figure S7.** miR-21-5p and/or miR-200a-depleted sEVs inhibit the proliferation and function of CD8$^+$ T cells.

A, B) qRT-PCR was used to detect the levels of miR-21-5p (A) or miR-200a (B) in sEVs derived from miR-21-5p- and/or miR-200a-depleteted SW620 cells.

C, D) Representative histogram of CFSE-labeled human peripheral CD8$^+$ T cells cocultured with differently pretreated macrophages. THP-1-derived macrophages were treated with miR-21-5p and/or miR-200a-depleted sEVs (C). IL-2 levels in these CD8$^+$ T cells were detected by ELISA (D). * $P < 0.05$, ** $P < 0.01$, *** $P < 0.001$.

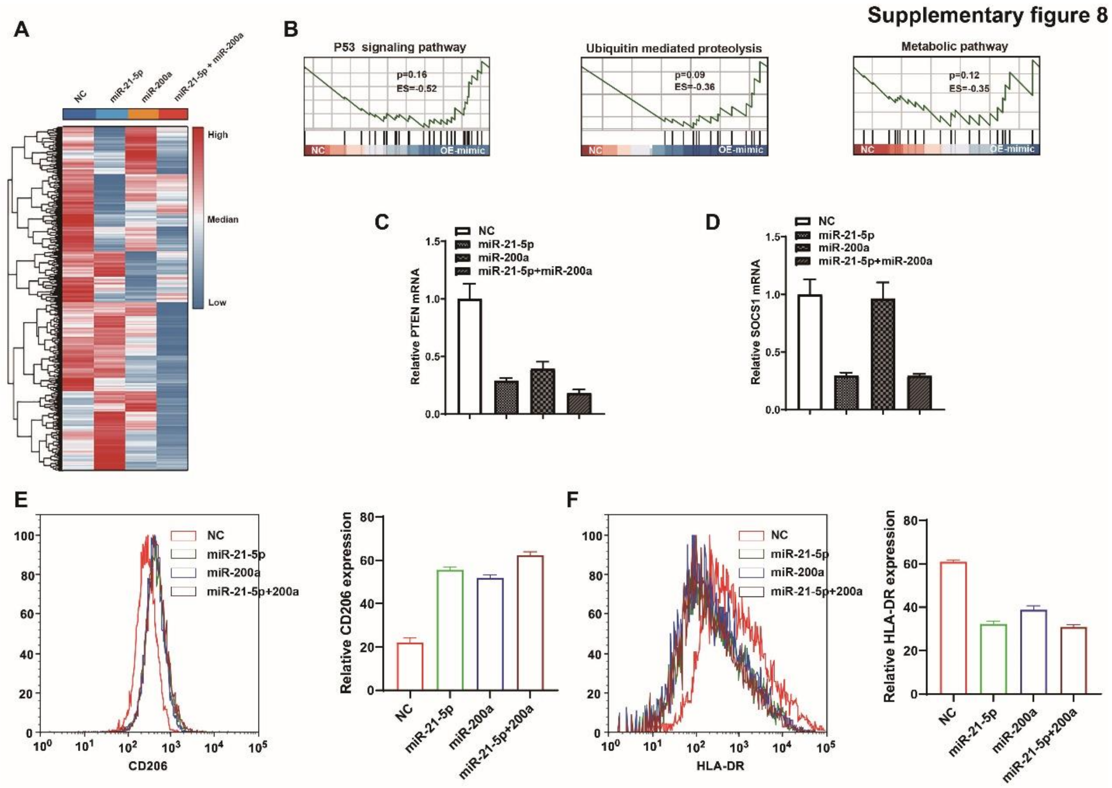


**Figure S8.** MiR-21-5p and miR-200a inhibit PTEN and/or SOCS1 expression and educate macrophages.

A, B) Gene expression profiles in macrophages transfected with NC-, miR-21-5p-, miR-200a- or miR-21-5p+miR-200a were detected using RNA sequencing (A). Gene set enrichment analysis (GSEA) was used to analyze the enriched pathways of the downregulated genes (B).

C, D) qRT-PCR was used to detect the expression of PTEN (C) and SCOS1 (D) in THP-1-derived macrophages transfected with miR-21-5p and/or miR-200a.

E, F) Ectopic expression of miR-21-5p and/or miR-200a induced CD206 expression and inhibited HLA-DR expression in macrophages. * $P$ < 0.05, ** $P$ < 0.01, *** $P$ < 0.001.

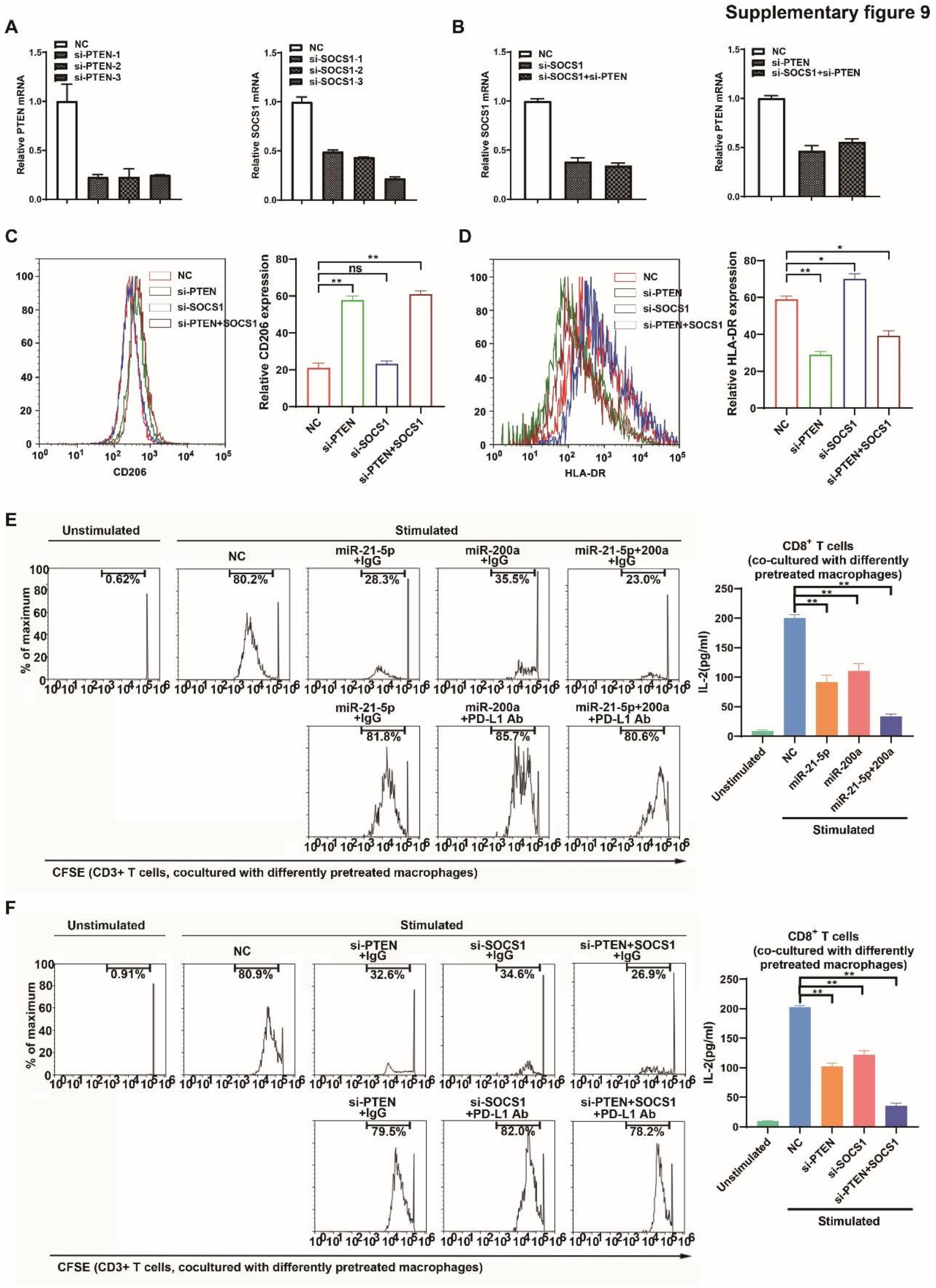


**Figure S9.** Ectopic expression of miR-21-5p and miR-200a or knockdown of PTEN and SOCS1 in macrophages inhibit the activities of CD8+T cells.

A, B) qRT-PCR was used to detect the knockdown efficiency of si-PTEN (A) and si-SOCS1 (B) in THP-1-derived macrophages.

C, D) Knockdown of PTEN and/or SOCS1 influence CD206 expression and HLA-DR

expression in macrophages.

E) Representative histogram of CFSE-labeled human peripheral CD8$^+$ T cells cocultured with differently pretreated macrophages. THP-1-derived macrophages were transfected with miR-21-5p and/or miR-200a and treated with anti-PD-L1 antibodies (10ug/ml). IL-2 levels in these CD8$^+$ T cells were detected by ELISA.

F) Representative histogram of CFSE-labeled human peripheral CD8$^+$ T cells cocultured with differently pretreated macrophages. THP-1-derived macrophages were transfected with si-PTEN and/or si-SOCS1. IL-2 levels in these CD8$^+$ T cells were detected by ELISA. * $P < 0.05$, ** $P < 0.01$, *** $P < 0.001$.

**Supplementary figure 10**

A

| Group | 293T sEVs (NC) | 293T sEVs (miR-21-5p) | 293T sEVs (miR-200a) | 293T sEVs (miR-21-5p+200a) | Total |
|---|---|---|---|---|---|
| CD8(low) PD-L1(low) | 1 | 1 | 1 | 0 | 3 |
| CD8(high)PD-L1(low) | 6 | 0 | 1 | 0 | 7 |
| CD8(low) PD-L1(high) | 0 | 5 | 5 | 6 | 16 |
| CD8(low) PD-L1(high) | 0 | 1 | 0 | 1 | 2 |
| Total | 7 | 7 | 7 | 7 | 28 |

Chi-Square=22.641 *p*<0.005

B

| CD8 protein levels | PD-L1 protein levels | |
|---|---|---|
| | Low | High |
| Low | 4 | 17 |
| High | 7 | 0 |

Sperman *r*=-0.23 *p*=0.015

**Figure S10.** The correlation between PD-L1+ macrophages and CD8+ T cells in the xenografts treated with CRC-derived sEV-miR-21-5p and/or sEV-miR-200a

A) sEV-miR-21-5p and sEV-miR-200a treatment significantly decreased the infiltration of CD8$^+$ T cells into tumor tissues.

B) The negative correlation between PD-L1 and CD8 protein levels in these xenografts.
