# Supplementary material for: Colorectal Cancer‐Derived Small Extracellular Vesicles Promote Tumor Immune Evasion by Upregulating PD‐L1 Expression in Tumor‐Associated Macrophages
Source: Adv Sci (Weinh). 2022 Jan 17;9(9):2102620. doi: 10.1002/advs.202102620 (PMC8948581; doi:10.1002/advs.202102620)
Supplement: Supplementary file 3 — Supporting Information [file ADVS-9-2102620-s003.pdf]

ADVANCED SCIENCE
Open Access

Supporting Information

for *Adv. Sci.*, DOI 10.1002/advs.202102620

Colorectal Cancer-Derived Small Extracellular Vesicles Promote Tumor Immune Evasion by Upregulating PD-L1 Expression in Tumor-Associated Macrophages

Yuan Yin, Bingxin Liu, Yulin Cao, Surui Yao, Yuhang Liu, Guoying Jin, Yan Qin, Ying Chen, Kaisa Cui, Leyuan Zhou, Zehua Bian, Bojian Fei, Shenglin Huang and Zhaohui Huang**

CRC cohorts from Affiliated Hospital of Jiangnan University

Variable	Univariable analysis			Multivariable analysis		
	HR	95%CI	*P* value	HR	95%CI	*P* value
Age						
＜60 or ≥60	1.2	(0.74-1.89)	0.092	1.52	（0.73-2.21）	0.231
Gender						
Male or Female	0.87	(0.65-1.74)	0.643	0.96	（0.83-1.63）	0.852
T stage						
1, 2, 3 or 4	1.32	(0.89-1.56)	0.031	1.46	（0.63-2.41）	0.151
N stage						
0, 1 or 2	1.54	(0.98-1.97)	0.052	1.76	（0.94-2.16）	0.125
M stage						
0 or 1	2.01	(0.99-4.81)	0.041	2.32	（0.87-4.27）	0.141
TNM Stage						
Ⅰ+Ⅱ or Ⅲ+Ⅳ	1.46	(0.98-2.21)	0.015	1.66	（0.95-2.54）	0.032
location						
Colon or Rectum	0.75	(0.75-1.01)	0.012	0.87	（0.66-0.97）	0.035
Tumor Size						
＜5cm or ≥5cm	0.93	(0.87-1.35)	0.091	0.99	（0.65-1.32）	0.152
$CD206^{+}CD274^{+}$						
low or high	1.45	(0.89-2.15)	0.013	1.61	(0.78-2.12)	0.031

TCGA-CRC

Variable	Univariable analysis			Multivariable analysis		
	HR	95%CI	*P* value	HR	95%CI	*P* value
Age						
＜60 or ≥60	1.5	(1.2-1.8)	0.00079	1.6	(1.3-2)	0.66
Gender						
Male or Female	0.88	(2.5-5.5)	0.51			
T stage						
1, 2, 3 or 4	3.1	(1.6-2.4)	8e-10	2.4	(1.5-3.7)	1.6e-05
N stage						
0, 1 or 2	2	(2.1-4.5)	5.5e-09	1.5	(1-2.1)	0.16
M stage						
0 or 1	3.7	(1.7-2.6)	6.1e-12	1.8	(0.79-4)	0.041
TNM Stage						
Ⅰ, Ⅱ, Ⅲ or Ⅳ	2.1	(0.61-1.3)	9.2e-11	1.1	(0.63-2.1)	0.00011
$CD206^{+}CD274^{+}$						
low or high	1.7	(0.7-1.6)	8.2e-09	1.42	(0.71-2.3)	0.02
